# Comparative (Within Species) Genomics of the *Vitis vinifera* L. Terpene Synthase Family to Explore the Impact of Genotypic Variation Using Phased Diploid Genomes

**DOI:** 10.3389/fgene.2020.00421

**Published:** 2020-05-05

**Authors:** Samuel Jacobus Smit, Melané Alethea Vivier, Philip Richard Young

**Affiliations:** South African Grape and Wine Research Institute, Department of Viticulture and Oenology, Stellenbosch University, Stellenbosch, South Africa

**Keywords:** terpene, *Vitis vinifera*, functional genomic analysis, gene annotation, carbocation cascade

## Abstract

The *Vitis vinifera* L. terpene synthase (*VviTPS*) family was comprehensively annotated on the phased diploid genomes of three closely related cultivars: Cabernet Sauvignon, Carménère and Chardonnay. *VviTPS* gene regions were grouped to chromosomes, with the haplotig assemblies used to identify allelic variants. Functional predictions of the *VviTPS* subfamilies were performed using enzyme active site phylogenies resulting in the putative identification of the initial substrate and cyclization mechanism of VviTPS enzymes. Subsequent groupings into conserved catalytic mechanisms was coupled with an analysis of cultivar-specific gene duplications, resulting in the identification of conserved and unique *VviTPS* clusters. These findings are presented as a collection of interactive networks where any *VviTPS* of interest can be queried through BLAST, allowing for a rapid identification of *VviTPS*-subfamily, enzyme mechanism and degree of connectivity (i.e., extent of duplication). The comparative genomic analyses presented expands our understanding of the *VviTPS* family and provides numerous new gene models from three diploid genomes.

## Introduction

Grapevine has an extensive domestication history that includes various non-*vinifera* hybridizations, resulting in high levels of heterozygosity (Minio et al., 2017). The sequencing of the *Vitis vinifera* cultivar Pinot Noir resulted in the first genome of a woody crop species (Jaillon et al., 2007; Velasco et al., 2007). Inbreeding of Pinot Noir simplified the genome to near homozygosity (93%) which facilitated sequencing of PN40024 (Jaillon et al., 2007). Concurrently a heterozygous clone of Pinot Noir, ENTAV115, was sequenced but difficulties in assembly of the heterozygous and highly repetitive regions resulted in a fragmented genome, limiting its usability (Velasco et al., 2007; Figueroa-Balderas et al., 2019). Continuous improvement over the last decade resulted in numerous assemblies and annotations of the PN40024 reference genome with the latest version (12X.v2 assembly and VCost.v3 annotation) improving the contig coverage and orientation by 14% over the previous assembly (12X.v0) and annotation (v1). However, 2.64 Mbp of contig sequences remain unmapped (chr. 00) while the orientation of numerous mapped contigs remain uncertain (Canaguier et al., 2017).

A combination of crossing (with close relatives as well as non-*vinifera* species) and millennia of propagation have resulted in the expansion of certain *V. vinifera* gene families. Of interest to this study are those linked to volatile organic compounds (VOC) that are often associated with aromatic cultivars. Terpenoids are known to modulate flavor and aroma profiles with monoterpenoids associated with floral and Muscat aromas while a spicy or pepper aroma, in certain wine styles, have been attributed to sesquiterpenoids (Siebert et al., 2008; Skinkis et al., 2008; Wood et al., 2008; Kalua and Boss, 2009; Black et al., 2015; Lin et al., 2019). The genetic potential of a cultivar to form terpenoids is highly variable and modulates the aromatic profile of the derived wine. Wine flavor and aroma is, however, complex and can be influenced by a multitude of factors that not only includes the cultivar but also vinification style, viticultural practices and extent of compound glycosylation (i.e., bound versus free volatiles) (Swiegers et al., 2005; Robinson et al., 2014; Hjelmeland et al., 2015; D’Onofrio et al., 2017). Terpenoids can furthermore be synthesized *de novo* by certain yeasts during fermentation, while other genera are known to liberate bound terpenoids by cleaving the glycosyl bonds (Carrau et al., 2005).

All terpenes consist of the C_5_ prenyl diphosphate building blocks isopentenyl diphosphate (IPP) and dimethylallyl diphosphate (DMAPP). These two building blocks arise from the 2-C-methyl-D-erythritol 4-phosphate (MEP) and mevalonate (MVA) pathways that are compartmentalized to the cytosol and plastids, respectively, although metabolic crosstalk between these pathways have been shown (Bloch et al., 1959; Lichtenthaler, 1999; Rohmer, 1999; Bick and Lange, 2003; Hemmerlin et al., 2003). Head-to-tail coupling of IPP and DMAPP results in elongated prenylated substrates that are characteristic to the various known terpene classes. Of particular interest in grapevine due to their volatile flavor and aroma properties, are the C_10_ mono- and C_15_ sesquiterpenes. Monoterpene biosynthesis proceeds through the MEP pathway with geranyl diphosphate (GPP) as the initial substrate with sesquiterpene biosynthesis proceeding through the MVA pathway using farnesyl diphosphate (FPP) and its isomer, nerolidyl diphosphate (NPP) as substrates (Davis and Croteau, 2000). The prenylated substrates can either be ionized or protonated to generate an initial reactive intermediate known as a carbocation, from which a concerted cascade of biochemical reactions proceeds. These reactions include ring-closures, hydride shifts, protonation and deprotonation events and various rearrangements. These cascades, therefore, result in various different carbocation intermediates being formed, subsequent to the initial, allowing for fairly conserved catalytic trajectories that define the enzyme mechanism (Cane, 1990; Davis and Croteau, 2000; Christianson, 2006; Wedler et al., 2015). Sesqui-TPS enzymes are more promiscuous in their product profile due to increased number of orientations that can arise from the added double bond of the FPP substrate, i.e., more possible carbocation intermediates. Enzyme promiscuity is known to be affected by subtle sequence variations in and around the enzyme active site that alter the product specificity or change the enzyme function completely (Li et al., 2013; Drew et al., 2015; Smit et al., 2019). By combining sequence homology of the active site with enzyme functions it is possible to predict how a TPS will interact with its substrate as well as predict the initial step in the carbocation cascade (Durairaj et al., 2019). The more than 40 characterized VviTPS enzymes from different *VviTPS* subfamilies therefore presents an opportunity for grapevine-specific functional predictions using sequence homology and a comprehensive understanding of TPS carbocation mechanisms.

Our current understanding of the grapevine terpene synthase *VviTPS* family is largely based on the PN40024 reference genome. This gene family is extensively duplicated with 152 loci and 69 putatively functional gene models, with the remaining loci being pseudogenes (Jaillon et al., 2007; Martin et al., 2010). However, nearly a third of the family is not mapped to a chromosome (i.e., found mapped to chr. 00), largely due to a lack of contiguity for genomic regions where *VviTPS* genes localize (Canaguier et al., 2017). Furthermore, cultivar-specific gene variations have been shown to impact enzyme function with subtle mutations altering the catalytic mechanism of the enzyme or, most often, rendering a gene non-functional (Drew et al., 2015; Dueholm et al., 2019; Smit et al., 2019).

The reference genome, being near-homozygous, can furthermore not be used to explore potential allelic differences. Allelic differences affecting VviTPS function have, however, been identified using the ENTAV115 heterozygous genome. Although this genome is highly fragmented, it still allowed for the identification of SNPs in *VviTPS24* that alters the catalytic mechanism from producing selinene-type sesquiterpenes to α-guaiene, the key precursors for synthesis of the rotundone sesquiterpene (associated with pepper aromas in wine) (Drew et al., 2015). Cultivar-specific *VviTPS* functions have been shown in a limited number of cultivars (Martin et al., 2010; Drew et al., 2015; Dueholm et al., 2019; Smit et al., 2019). Extrapolating this to the more than 6000 grapevine accessions planted worldwide (This et al., 2006) suggests extensive *VviTPS* diversity, with the PN40024 genome sequence likely representing only a fraction of the genetic potential.

**FIGURE 1:**
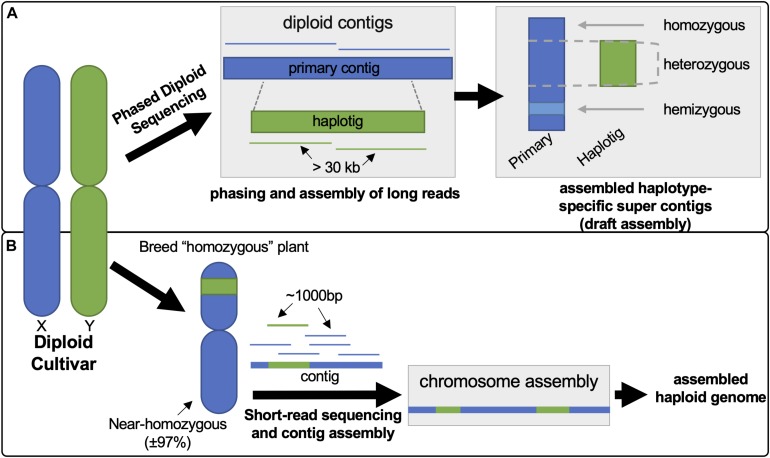
Illustration contrasting the differences in approach between phased diploid **(A)** and short-read sequencing **(B)** to generate the diploid and PN40024 reference genomes, respectively. **(A)** PacBio long-reads (> 30 kb) undergo phasing during the assembly, using FALCON-UNZIP, resulting in the generation of a pseudomolecule known as a primary contig. This primary contig region represents both possible haplotypes. The phasing algorithm identifies reads with high heterozygosity to call an alternate genomic region (haplotig) which is assembled separately allowing for the generation of haplotype regions. **(B)** The reference genome was generated from an inbred clone of Pinot Noir (PN40024), reducing genomic complexity to near homozygosity. Assembly of the short-reads, generation of contigs and subsequent mapping/assembly to chromosomes lead to the PN40024 reference genome.

The recently available draft diploid genome assemblies for grapevine provide extensive new genomic information that can be utilized to explore cultivar-specific *VviTPS* variation to understand structure-function relationships (i.e., gene-protein-terpene) for terpene biosynthesis. In this study Cabernet Sauvignon (CS), Carménère (CR), and Chardonnay (CH) were selected for this purpose as they were sequenced and assembled using the same technology: Pacific Biosciences Single Molecule Real Time Sequencing (PacBio-SMRT) sequencing with FALCON-UNZIP phased assembly (Chin et al., 2016; Roach et al., 2018; Minio et al., 2019). The PacBio-SMRT platform allows for long-read sequencing (> 30 kb), resulting in highly contiguous reads that are easier to assemble, but with a greater error rate (7–15%) than short-read sequencing (Rhoads and Au, 2015). The latter limitation is, however, overcome by the greater read-depth (> 115X versus 12X for the reference genome) (Chin et al., 2016; Figueroa-Balderas et al., 2019). Phased assembly with FALCON-UNZIP allowed for haplotype resolution, resulting in affectively two assemblies: the primary assembly, consisting of highly contiguous pseudo-molecules that contain both haplotypes, and the haplotig assembly, consisting of shorter phased reads that represent alternate alleles (Chin et al., 2016; Minio et al., 2017). The differences in assembly approach between the latest diploid grapevine genome and the PN40024 reference genome is illustrated in Figure 1. The diploid genomes of CS, CR and CH are highly contiguous and more complete than the PN40024 reference genome (N50 of 0.94–2.17 Mbp versus 0.103 Mbp). The phased diploid genomes therefore allow for genomic sequence data that captures homo- and heterozygous gene regions as well is hemizygous regions (gene regions unique to a haplotype) (Jaillon et al., 2007; Chin et al., 2016; Roach et al., 2018; Minio et al., 2019). The diploid genomes sizes are, however, inflated (more than double the haploid genomes) due to haplotype regions being missed in regions of high heterozygosity, resulting in the haplotypes being incorrectly assigned to the primary assembly (Minio et al., 2017; Figueroa-Balderas et al., 2019).

Focusing on the *VviTPS* family, the aim was to evaluate and correct gene models, where necessary, and then explore the extent of haplotype and genotype variations using the phased diploid draft assemblies. An in-depth analysis of the three genomes ultimately resulted in a significant extension of current knowledge on the *VviTPS* family; which includes chromosome groupings, functional prediction (which includes TPS-subfamily, initial substrate and cyclization mechanisms), cultivar-specific duplication analysis and identification of conserved VviTPS functions. Interactive networks were constructed for gene duplication and genotype/haplotype variations, making the data easily accessible. These networks can be queried using BLAST and all relevant VviTPS information interactively accessed in the respective networks.

## Methodology

### Genome Assemblies and Annotations Utilized

Genomes for *V. vinifera* cultivars (Jaillon et al., 2007; Chin et al., 2016; Minio et al., 2017, 2019; Roach et al., 2018) listed in Table 1 where downloaded from the listed repositories. PN40024 12X.v2 assembly and VCost.v3 (V3) annotation was used as the reference genome (Jaillon et al., 2007; Canaguier et al., 2017). The GFF3 annotation for the terpene synthase family^1^ was used for *VviTPS* positioning on the reference genome. PN40024 *VviTPS* sequences identified and curated by Martin et al. (2010) were retrieved from FLAGdb + + (Dèrozier et al., 2011).

**TABLE 1:** Genome assemblies and annotations utilized.

Genome	Assembly type	Annotation version	Repository
PN40024 12X.v2 (PN)	Haploid	VCost.v3	https://urgi.versailles.inra.fr/Species/Vitis
Cabernet sauvignon (CS)	Diploid	V1	http://cantulab.github.io/data.html
Carménère (CR)	Diploid	V1	http://cantulab.github.io/data.html
Chardonnay (CH)	Diploid	V1	https://doi.org/10.5281/zenodo.1480037

The domestication history of these cultivars was inferred by using the *Vitis* International Variety Catalog^2^ and domestication histories described by Myles et al. (2011) and Minio et al. (2019).

### Identification and Annotation of *VviTPS* Gene Regions on the Diploid Genomes

The Exonerate tool (Slater and Birney, 2005) was used to identify VviTPS-like regions on the primary and haplotig assemblies of the respective diploid genomes (Table 1). PN *VviTPS* gene models served as query sequences with the exonerate parameters set to the est2genome model, percentage of the maximal score set at 90% and intron size limited to 3000 bp. The est2genome model parameter employs a gapped alignment algorithm of all *VviTPS* reference sequences to query all primary contig and haplotig sequences for the presence of a *VviTPS*-like gene region. A detailed explanation of the Exonerate analysis can be found in Supplementary Data Sheet 1. Exonerate computations were performed using the Stellenbosch University Central Analytical Facilities’ HPC2: http://www.sun.ac.za/hpc. Exonerate-gff outputs were annotated on the respective genome contigs and manually curated with CLC Main Workbench 7 (CLC Bio-Qiagen, Denmark) to identify hit regions with the greatest coverage and highest mapping score.

The identified gene regions were compared with the computational annotations reported for the respective genomes in Table 1. Each identified gene region was assigned a unique accession consisting of a two-letter cultivar code (Table 1) followed by a sequential TPS number. When automated annotations for the respective genomes (Table 1) where congruent with the annotation generated in this study, the annotation specific locus ID was maintained as the parent ID in the annotation file. Annotated coding sequences of these congruent regions were maintained as far as possible, but manual correction of several gene regions was necessary with such corrections noted in the annotation file. Gene regions lacking a parent ID indicates a newly annotated region. Partial genes were noted when there were four or less exons and a lack of start and/or stop codons. Genes were considered to be complete if a start and stop codon was present at the terminal ends and the exon number was greater than four. Complete genes were subsequently evaluated for the presence of a reading frame, with genes lacking a full-length open reading frame (fl-ORF) tagged as disrupted (d-ORF), with the disruption being either a premature stop, or a frameshift (insertion-deletion) mutation.

Protein sequences were derived for the fl-ORF’s and the terpene synthase N-terminal (PF01397) and C-terminal (PF03936) domains predicted using the PFam Domain Search function of CLC Main Workbench 7 (CLC Bio-Qiagen, Denmark). The motif search function CLC Main Workbench 7 was used to identify motifs characteristic to TPS proteins (Starks, 1997; Williams et al., 1998; Rynkiewicz et al., 2002; Gao et al., 2012; Durairaj et al., 2019).

### Putative Identification of Duplicated Gene Regions

A BLASTn alignment (Altschul et al., 1997; Camacho et al., 2009) of complete gene regions for each cultivar was performed and duplications identified by calculating the identity (*I*′) using the formula described by Li et al. (2001), with *I* being the number of identities and gaps, *n* the aligned length and *L* the total length of the query and subject sequences, respectively. For BLASTn analyses of primary-to-primary and haplotig-to-haplotig complete genes, an *E*-value of 1e-5 was used, with the maximum number of alignments (max-hsps) limited to 5 and number of aligned sequences (max-target-seqs) set to 10. The latter two parameters were set to 1 when haplotigs-to-primary alignments were performed.

### Rapid Assembly of Contigs

Chromosome positions of *VviTPS*-like gene regions were inferred by mapping all *VviTPS* containing contigs to the PN reference genome using rapid reference-guided assembly (RaGOO) (Canaguier et al., 2017; Alonge et al., 2019). The RaGOO parameters for chimera breaking (-b), structural variant calling (-s) and a gap padding (-g) of 200 were used with unplaced contigs not assembled to a random chromosome. The random pseudo-molecule (chr. 00) of the reference genome was not included for RaGOO assemblies. The output of this cultivar-specific all-against-all assembly was used to group contigs according to their highest scoring PN40024 chromosome, followed by chromosome specific contig assembly. The respective RaGOO outputs were visualized using the contig alignment function of the Alvis tool (Martin and Leggett, 2019).

### Functional Annotation of *VviTPS* Genes

Multiple sequence alignments (MSA) and phylogenetic tree constructions were performed in the CLC Main Workbench 7 (CLC Bio-Qiagen, Denmark). For nucleotide alignments the ClustalO algorithm was used while the MUSCLE algorithm was used for protein sequences. Phylogenetic trees were constructed with UPGMA, Jukes-Cantor as distance measure and 100 bootstrap replicates (Jukes and Cantor, 1969; Edgar, 2004a, b). MSA’s were performed at the nucleotide level using the 152 *VviTPS* gDNA and mRNA sequences predicted by Martin et al. (2010) as reference. Phylogenetic position relative to PN40024 gDNA sequences were used to group gene regions into TPS-subfamilies (Bohlmann et al., 1998; Martin et al., 2010) with the eulerr R package (R Core Team, 2013; Larsson, 2019) used to visualize the data.

Protein sequence phylogenies with characterized grapevine TPSs (Supplementary Table 1) were used to group proteins into TPS-subfamilies. For the TPS-a subfamily, the active site region was identified as described by Durairaj et al. (2019) and aligned as described earlier. This active site phylogeny and the Database of Characterized Plant Sesquiterpene Synthases (Durairaj et al., 2019) was used to divide TPS-a members into groups based on their parent cation and first cyclization. For the TPS-b subfamily a similar approach to Durairaj et al. (2019) was applied where only the active site region between the C-terminal metal binding motifs, if present, were aligned. The product profiles of TPS-b members (Martin et al., 2010) were used to predict a mono-TPS reaction mechanism (Williams et al., 1998; Davis and Croteau, 2000; Schwab et al., 2001; Hyatt et al., 2007; Schwab and Wüst, 2015; Xu et al., 2018) and categorize proteins according to their initial carbocation intermediate (terpinyl or linalyl cation). The latter was further subcategorised by considering whether or not quenching occurs before deprotonation. The TPS-g subfamily was subcategorised using the full-length protein alignment and phylogenetic position relative to functional proteins (Martin et al., 2010).

### Finding Homologous Proteins Between Cultivars

The cluster function of MMseqs2 (Steinegger and Söding, 2017) was used for all-against-all clustering of proteins with the following parameters: bidirectional alignment coverage mode with a minimum coverage of 85%, minimum sequence identity of 75%, *E*-value of 1e-5 and greedy clustering (cluster-mode 2). Representative sequences from the clustering were extracted as described in Supplementary Data Sheet 2.

### Network Construction

Cytoscape v3.7.2 (Shannon et al., 2003) was used to construct all networks presented in this study with the data generated from the aforementioned methodologies used for node and edge metadata.

## Results

### Relatedness of the Genomes

The domestication history of grapevine (Myles et al., 2011) and available pedigree information (Maul and Töpfer, 2015) shows that CR and CS have a common parent while Pinot Noir is a parent to CH. All cultivars share Traminer as an ancestor. The relatedness (pedigree) of cultivars used for genomes discussed in this study is shown in Supplementary Figure 1.

### Diploid Genome VviTPS-Like Gene Regions

Nearly all of the diploid contigs annotated with a *VviTPS* could be assigned to a reference chromosome using RaGOO, with the exception of 1 CS and 2 CR contigs. The position of the mapped contigs were congruent to *VviTPS* containing chromosomes of the reference genome (Martin et al., 2010). The RaGOO grouping scores per chromosome (Supplementary Table 2) ranged between 53% and 97% with an average of 75%, indicating that the contigs could be placed on a chromosome with an acceptable level of confidence. However, the exact position on a chromosome could not be accurately estimated, as evident by the location scores, reflecting a low level of collinearity to the reference genome (Supplementary Table 2). Contig alignments to the reference genome using Alvis (Supplementary Data Sheet 3) clearly illustrates the extent of discontiguity when mapping the phased diploid contigs to the reference genome.

**FIGURE 2:**
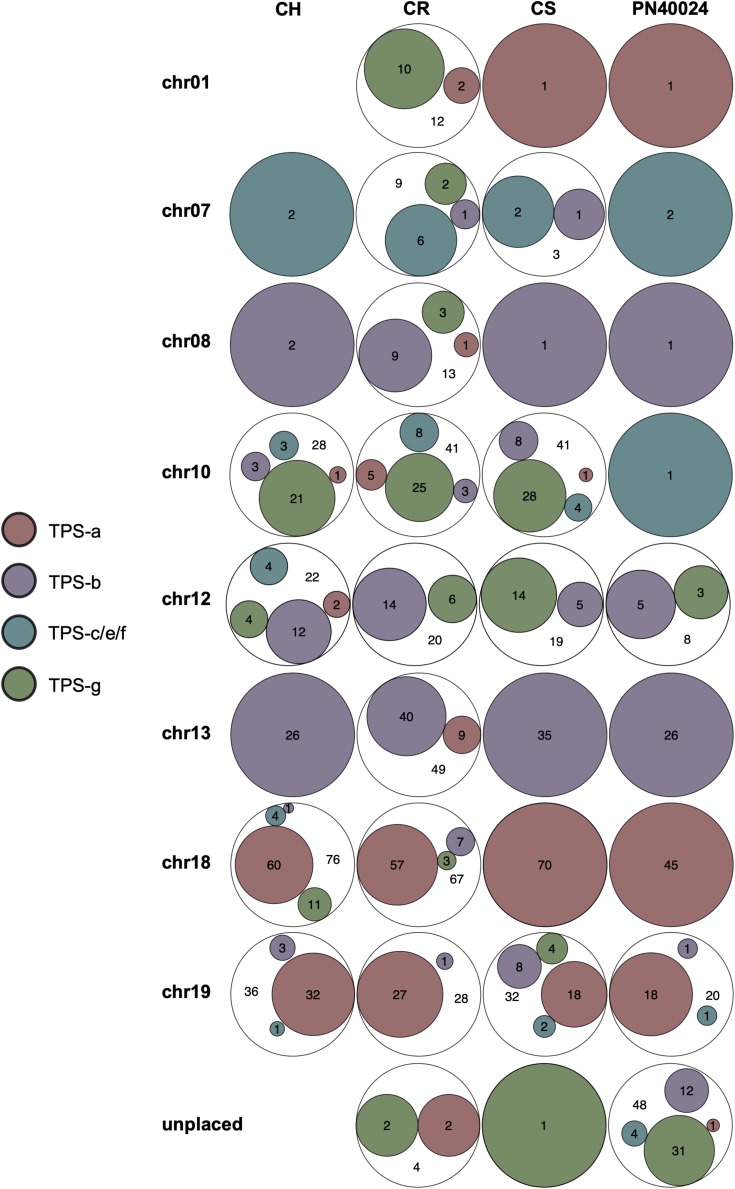
Euler diagrams summarizing the chromosome specific distribution of *VviTPS*-subfamilies for each of the diploid genomes: Cabernet Sauvignon (CS), Carménère (CR), and Chardonnay (CH) as well as the Martin et al. (2010) annotation of PN40024 (PN). The legend shows the different *VviTPS* subfamilies, proportionally sized within the Euler diagrams to reflect the total number of *VviTPS*-like gene regions per cultivar and chromosome.

The Euler graphs in Figure 2 show *VviTPS* subfamily members per chromosome for the diploid assemblies with PN40024 as a reference. Despite the latest assembly improvements for PN40024, a large number *VviTPS* genes are yet to be assembled to a chromosome, reflected by the “unplaced” genes in Figure 2. The diploid assemblies showed an inverse proportional relationship between unplaced and chr. 10 genes relative to PN40024, indicating that long read sequencing has overcome, to a large extent, the unresolved location of chr. 10 *VviTPS* genes. 28 *VviTPS* genes for CH and 41 for CR and CS, respectively, were placed on chr. 10, compared to a single gene on PN40024 (Martin et al., 2010; Canaguier et al., 2017). The majority of these genes are homologous to members of the PN40024 TPS-g subfamily, as illustrated by the gDNA phylogeny in Supplementary Figure 2. Furthermore, CR had more than three times the number of genes on chr. 01, 07 and −08 than CS, CH or PN. In agreement with the reference genome, the majority of TPS-a genes are located on chr. 18 and −19 with nearly all TPS-b genes on chr. 13.

**FIGURE 3:**
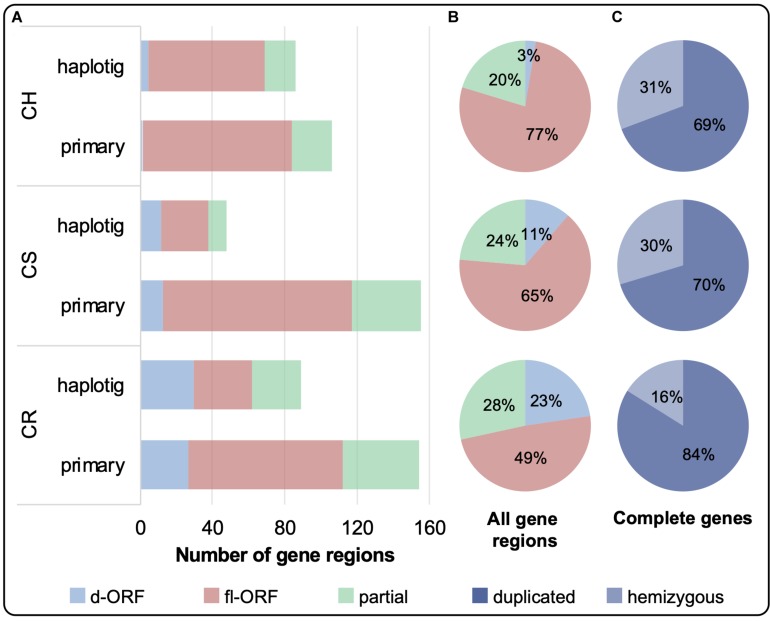
**(A)** The total number of *VviTPS*-like gene regions on the primary contigs and haplotigs is shown for the draft diploid genomes of Cabernet Sauvignon (CS), Carménère (CR), and Chardonnay (CH). **(A)** The total number of *VviTPS*-like gene regions were further classified by the type of open reading frame (ORF): disrupted (d-ORF) contain frameshifts and/or premature stop codons that render the gene non-functional; full-length (fl-ORF) are predicted to be functional; and partial genes that have four less exons (i.e., pseudogenes). The combined percentage distribution of these ORFs across the haplotypes is shown in **(B)**. The percentage of complete gene regions, the sum of fl-ORF and d-ORF, that are duplicated (degree of similarity (*I’)* > 80%) or hemizygous is shown in **(C)**.

The distribution of complete and partial gene regions on the primary and haplotig assemblies is shown in Figure 3A. Complete gene regions were sub-categorized into fl-ORF or d-ORF, with the latter representing regions that can also be considered as pseudogenes. Although CR had the greatest number of *VviTPS*-like regions (243), only 49% of these regions encode for a putative fl-ORF, shown in Figure 3B, with 84% of the complete genes being duplicated (Figure 3C). CS and CH had a similar number of *VviTPS*-like regions (203 and 192, respectively), with CH showing the greatest proportion of fl-ORF (77%) of all three cultivars (Figure 3B). CS and CH *VviTPS* families are also extensively duplicated, however, ∼30% of their complete *VviTPS* genes were hemizygous (Figure 3C).

**FIGURE 4:**
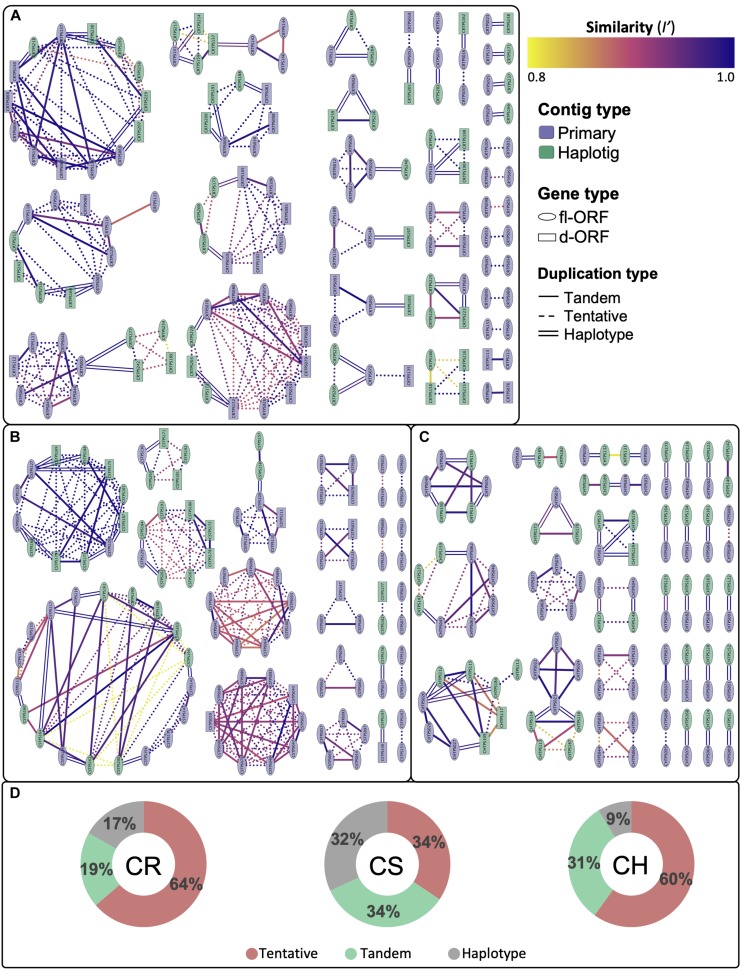
Cytoscape network illustrating the connectedness and degree of similarity (*I’*) for duplicated complete genes for **(A)** Carménère (CR), **(B)** Cabernet Sauvignon (CS) and **(C)** Chardonnay (CH). Nodes represent *VviTPS* genes and are connected by edges, signifying homology of *I’* > 80%. Complete genes are grouped into those with a full-length or disrupted open reading frames (fl-ORF or d-ORF) for the cultivar-specific haplotypes. The type of edge interactions are further categorized as tandem duplicates if the gene is present on the same contig; haplotype duplications are on primary contigs and haplotigs that localize to the same chromosome and were inferred from RaGOO assemblies to PN40024; with tentative duplications showing genes with high homology that cannot be defined by the two previous groupings. The total percentage contribution of these groupings is shown in **(D)**.

Despite the diploid genomes being unassembled, the size and contiguity of the phased diploid contig assemblies allowed for the extent of gene duplications to be investigated, as illustrated by the cultivar specific networks in Figures 4A–C. Gene regions with an identity score (*I*′) greater than 80% were considered to be duplicated with those localizing to the same contig considered to be tandem duplicates. Tentative duplications show genes that are not on the same contig (i.e., possible genome wide duplications). Supplementary Figure 3 shows an alternative node coloring for the aforementioned figure, illustrating their chromosomal localization. The duplication distribution in the edge interactions graph (Figure 4D) gives an estimation of the homozygosity for each cultivar, with CS showing the greatest percentage (32%) of haplotype edge connections, i.e., potential allelic variants.

### Functional Annotation of the *VviTPS-a* and *-b* Subfamilies

Protein sequences derived from fl-ORFs and subsequent phylogenetic similarity to known functional VviTPS enzymes clearly separate the proteins into subfamilies, illustrated in Supplementary Figure 4. The VviTPS-a, -b and -g subfamilies represent the majority of putative proteins and were subsequently analyzed in a family specific manner to predict their function.

**FIGURE 5:**
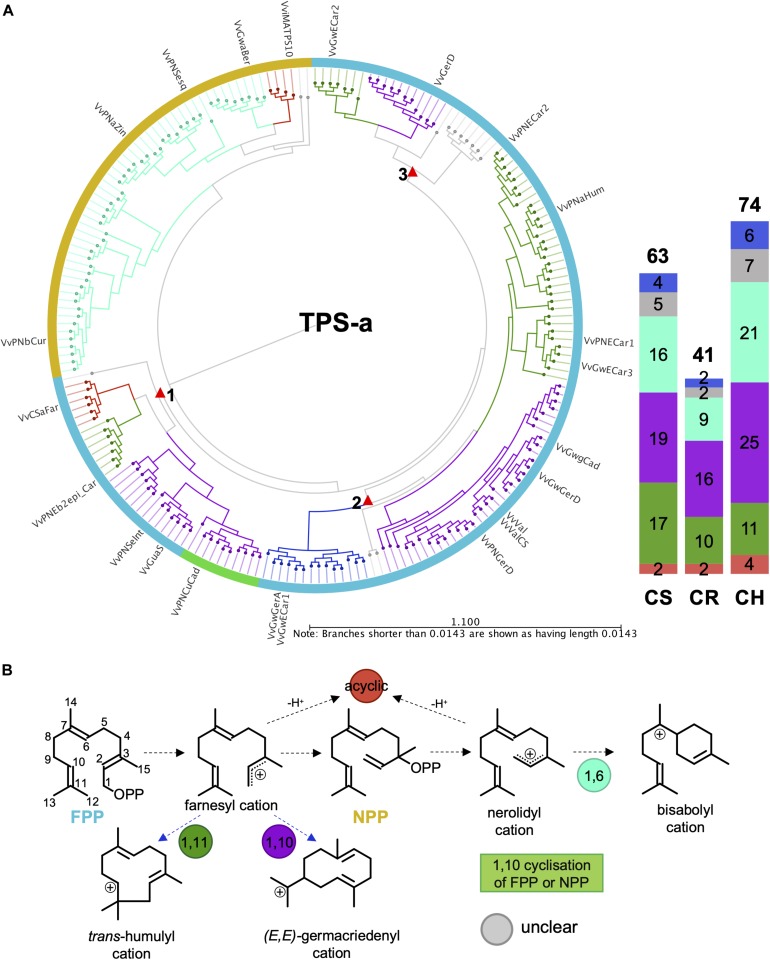
**(A)** Active site phylogeny of putative VviTPS-a proteins from the diploid genomes and functionally characterized enzymes (outer labels) were used to predict the initial substrate (outer colored ring) and cyclization mechanism (branch color). The red triangles indicate subclades for enzymes that utilize farnesyl diphosphate (FPP) as initial substrate, shown by the blue outer ring. The yellow outer ring represents enzymes that utilize nerolidyl diphosphate (NPP) as initial substrate. Both FPP and NPP can be ionized or protonated to form the initial carbocation intermediates indicated in **(B)**. Branches in **(A)** are colored according to these initial carbocation cyclization mechanisms and the subsequent carbocation formed, shown in **(B)**. Deprotonation of the initial carbocation result in the formation of acyclic sesquiterpenes, as shown in **(B)**.

The VviTPS-a subfamily separates into three major groups based on the initial substrate (FPP and/or NPP) utilized, illustrated in Figures 5A,B. Two acyclic subgroups were associated with each of these substrates. With the exception of the acyclic sesquiterpenes, all enzymes that use NPP as sole substrate will proceed through an initial 1,6-cyclization of the nerolidyl cation (Davis and Croteau, 2000). Reactions mechanisms that proceed from FPP formed three distinct clades, indicated by the red triangles, with each clade showing a group for 1,10- and 1,11-cyclizations. Acyclic sesquiterpenes and those that require 1,11-cyclization showed commonality in clade 1 that is distinct from the 1,10-cyclization group. Clade 2 showed three distinct groups with a unique subgroup consisting of both 1,10 and 1,11-cyclization enzymes. The third clade had a number of enzymes that could not be definitively placed into a cyclization group but, as with clade 2, showed clear separation between the 1,10 and 1,11 cyclization mechanisms. The putatively functional *VviTPS-a* genes for each cultivar ranged between 41 and 74, as illustrated by the bar graph in Figure 5. Furthermore, the number of genes associated with the respective carbocation cascades (Figure 5B) differs between cultivars. The 1,10 and 1,11 cyclization of FPP represents the majority of reaction mechanisms in all cultivars. Enzymes predicted to form acyclic sesquiterpenes were limited to between 2 and 4, while 1,6 cyclization of NPP represents less than a third of the predicted mechanisms.

**FIGURE 6:**
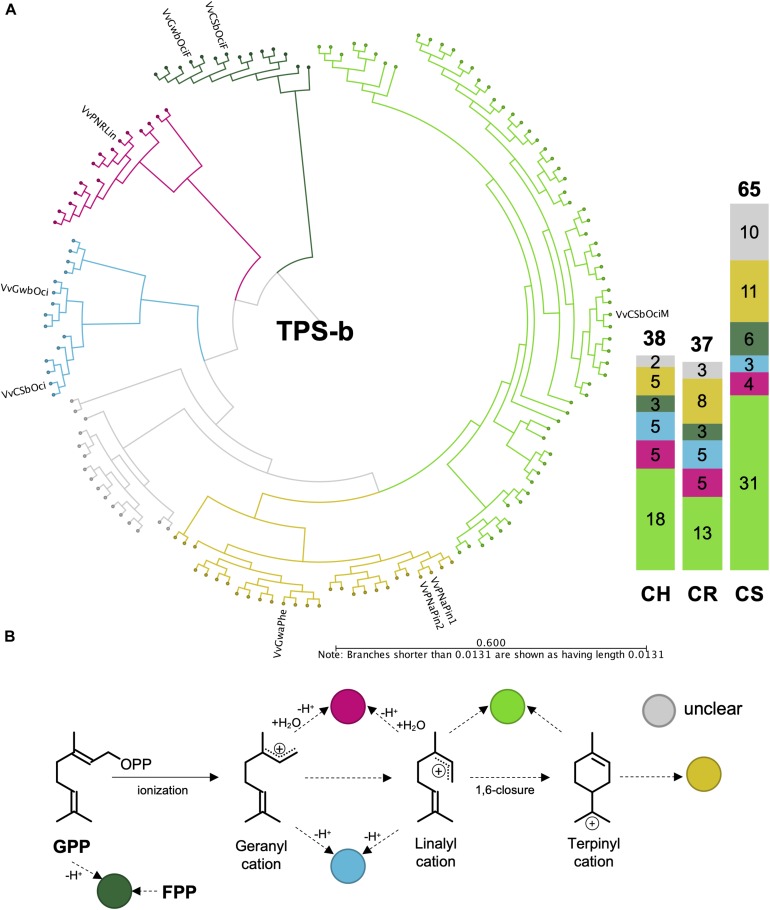
**(A)** Active site phylogeny of putative VviTPS-b proteins from the diploid genomes and functionally characterized enzymes (outer labels) were used to predict the carbocation cascades that proceed from geranyl diphosphate (GPP), as shown in **(B)**. The dark green branches represent enzymes that can utilize both GPP and farnesyl diphosphate (FPP), functioning as single product enzymes to form the monoterpene ocimene or the sesquiterpene (*E,E*)-alpha-farnesene, respectively. The pink branch is represented by a single product enzyme that proceeds through either the geranyl and/or linalyl cations, with a concerted protonation and deprotonation cascade to form the acyclic monoterpene alcohol limonene. Deprotonation of the geranyl and/or linalyl cations will result in the formation of the acyclic monoterpene ocimene, shown by the blue branch. The light green branch is represented by a multiproduct monoterpene synthase with a cascade proceeding from the linalyl cation to produce acyclic terpenes with a 1,6 ring-closure required to form the terpinyl carbocation and subsequent cyclic terpenes. The yellow branch represents multiproduct enzymes that produce only cyclic monoterpenes, with the gray branch representing a clade without a functional enzyme.

Although the VviTPS-b subfamily utilizes a single substrate for monoterpene biosynthesis, enzymes could still be grouped into distinct reaction mechanisms, illustrated in Figures 6A,B where the cyclic reaction mechanism is referred to as TPS-b Type I while the acyclic mechanism is referred to as TPS-b Type II. Type II enzymes however, formed three distinct clades, of which two are for the single product enzymes associated with linalool (red branch) and ocimene biosynthesis (blue branch), VvPNRLin and VvGwBOci/VvCSbOci, respectively (Martin et al., 2010). The third Type II clade (light green branch) is represented by a single functional enzyme (VvCSbOciM) that produces 98% acyclic monoterpenes, (*E*)-beta-ocimene and myrcene, and minor amount of the cyclic monoterpene pinene (Martin et al., 2010). This clade is also the largest in all three cultivars, as shown by the bar graph in Figure 6, and is closely related to a clade of multiproduct Type I mono-TPS enzymes (yellow branch). The phylogenetic distribution and predicted reaction mechanisms therefore show that the majority of mono-TPS genes will produce both cyclic and acyclic monoterpenes.

### Functional Annotation of the *VviTPS-g* Subfamily

**FIGURE 7:**
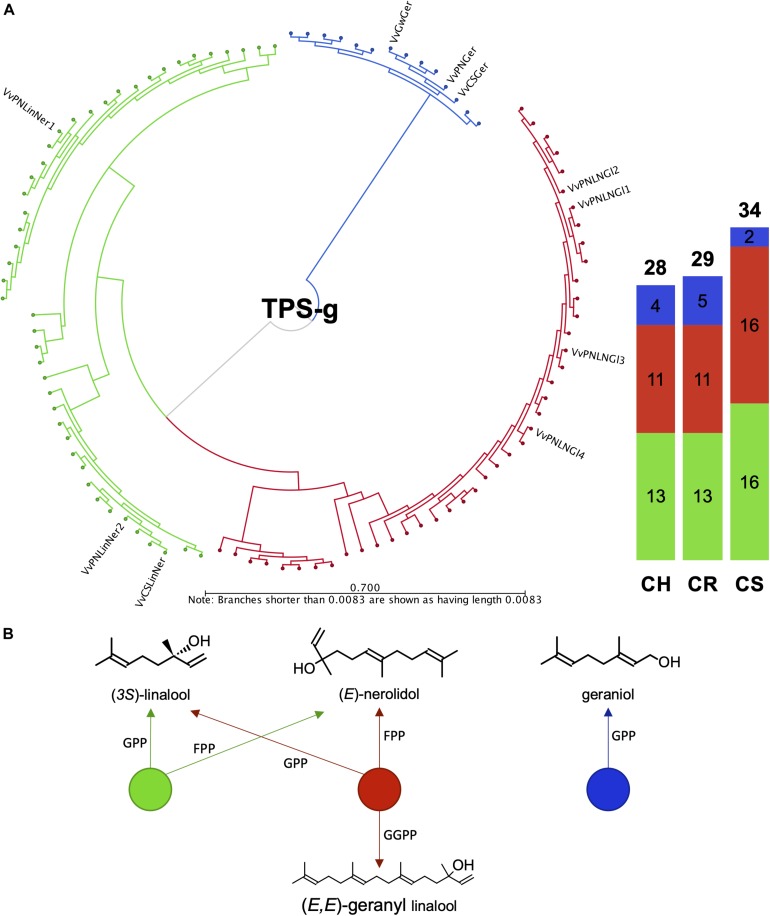
**(A)** Full length amino acid sequence phylogeny of putative VviTPS-g proteins identified for the diploid genomes and functionally characterized VviTPS-g members (outer label). Branches reflect the enzyme product profile and substrate specificity shown in **(B)**. The green clade consists of single product enzymes that utilize both geranyl diphosphate (GPP) and farnesyl diphosphate (FPP) to produce the acyclic monoterpene alcohol (3*S*)-linalool or acyclic sesquiterpene alcohol (*E*)-nerolidol, respectively. The red clade represents enzymes that in addition to the mechanism shown in green, accept geranyl geranyl diphosphate (GGPP) to produce the acyclic diterpene alcohol (*E,E*)-geranyl linalool. The blue clade represents enzymes that only accept GPP to form the acyclic monoterpene alcohol geraniol.

It was previously shown that the *VviTPS-g* family is expanded in grapevine, forming three distinct clades that separate according to the product profiles of *in vitro* characterized TPS-g enzymes (Martin et al., 2010). Those that accept only GPP as substrate to form geraniol formed a distinct clade with the multi-substrate enzymes forming the other two clades, as shown in Figures 7A,B. Annotation of this subfamily to a large extent resolved the current lack in chromosome mapping of this family (Figure 2, Supplementary Figure 2 and Supplementary Data Sheet 3). Despite the various improvements of the reference genome, chr. 10 remained difficult to assemble, with inbreeding of PN40024 not being able to reduce the extent of heterozygosity. The lack of sufficient resolution for this chromosome therefore resulted in highly discontiguous mapping of diploid contigs to PN40024 chr. 10. This discontiguity resulted in only a single *TPS-g* member being represented on chr. 10 of PN40024 (Martin et al., 2010), with the remaining members being unplaced (i.e., chr. 00). The results presented for the draft diploid genomes therefore provide new chromosome specific information of the *VviTPS-g* subfamily.

CS had 28/34 *VviTPS-g* members that mapped to chr. 10, of which 14 were located in a 262 kb region of the primary contig VvCabSauv08_v1_Primary000201F. Seven of the genes in this cluster were predicted to be functional and are highly connected to genes from seven different haplotigs, all mapping to chr. 10 (Figures 4B, 7). Furthermore, the *VviTPS* gene order of the primary contig was dissimilar to the haplotigs with large size differences for the intergenic regions, indicating a high level of heterozygosity for this chromosome (Figure 2, Supplementary Figure 2, and Supplementary Data Sheet 3). CR had a similar sized *VviTPS-g* family on chr. 10, localizing to two different primary contigs with almost no contiguity to PN-chr. 10 (Figure 2, Supplementary Figure 2, and Supplementary Data Sheet 3). It was evident from the haplotig to primary contig mappings that chr. 10 is also highly heterozygous for CR. CH, was the exception with 17/28 *VviTPS-g* members mapping to chr. 10 (Figures 2, 7, Supplementary Figure 2, and Supplementary Data Sheet 3). All seventeen are located on a single contig, connected as tandem duplications in Figure 4C, suggesting that it is more homozygous for *VviTPS-g* members on chr. 10. As with the other two genomes, this region was highly discontiguous to the reference genome (Supplementary Data Sheet 3).

### Comparative Genomics Using Interactive Networks

**FIGURE 8:**
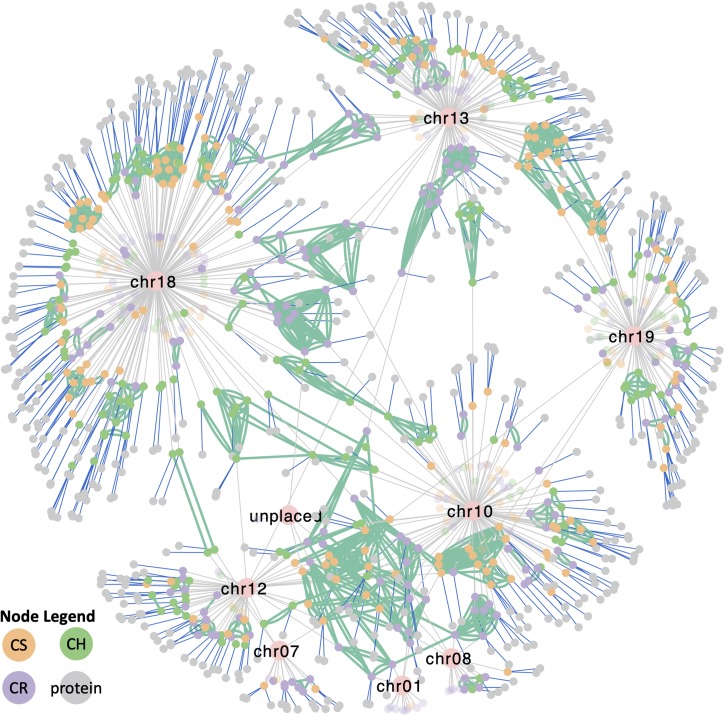
Cytoscape network representing all metadata represented in Figures 2–7 of the respective *VviTPS* gene families for Cabernet Sauvignon (CS), Carménère (CR), and Chardonnay (CH). Nodes represent *VviTPS* genes, colored according to cultivar with the lighter shade showing partial genes. Gray edges show the chromosome mapping (pink nodes), with green edges showing genes with high homology (degree of similarity (*I’*) > 80%). Green edges connecting nodes of the same color reflect within cultivar homologs (i.e., duplicated genes) while edge connections between different colored nodes show cross-cultivar homologs. Blue edges show putative proteins associated with a gene (i.e., predicted to be functional). An interactive version of this network to access the metadata is available online.

To understand the complexity of the *VviTPS* family, an integrated view of all the components that influence the different subfamilies is required. The network in Figure 8 shows the *VviTPS* containing chromosomes, gene duplications and putative proteins for the three diploid genomes. Contig nodes were excluded from the visualization but can be accessed in the interactive network online. The three major *VviTPS-*containing chromosomes, namely chr. 13, −18 and −19 show extensive duplications on the respective chromosomes with few shared between chromosomes. Although the remaining chromosomes, excluding chr. 10, have few *VviTPS* genes, it is evident that they are extensively connected between chromosomes, specifically the multi-substrate *TPS-g* family of chr. 10.

**FIGURE 9:**
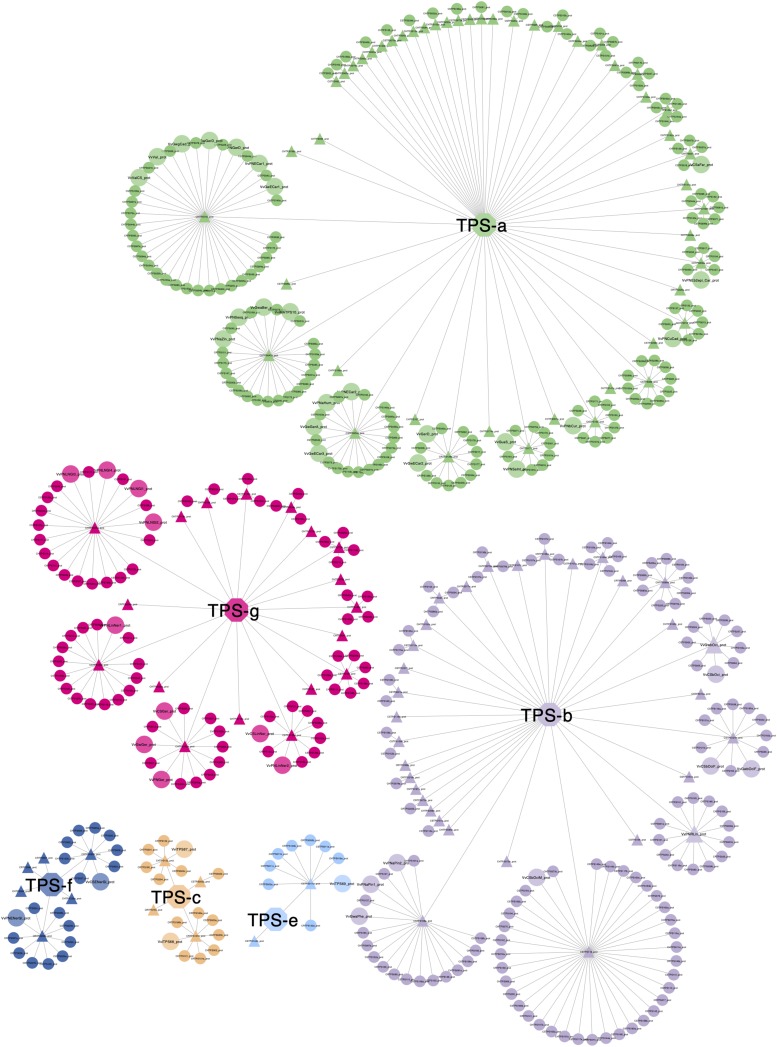
Representative proteins (triangles) show clustering of conserved proteins per *VviTPS* subfamily (central nodes with subfamily name). An absence of connections indicate that the sequence is unique. Enlarged circular nodes are functionally characterized enzymes. The representative sequences serve as target sequences through BLASTp in order to query any TPS of interest to identify mechanistically conserved enzyme clusters. To access the metadata through BLASTp refer to the network available online and the accompanying help document, as described in the supplementary data.

An all-against-all clustering of diploid genome putative VviTPS proteins and functionally characterized proteins is shown in Figure 9. The network consists of 533 proteins of which 44 are functionally characterized (Lücker et al., 2004; Martin et al., 2009, 2010; Drew et al., 2015; Smit et al., 2019), sized and shaded in Figure 9. To date no *VviTPS-c* or *-e* members have been characterized, therefore the three predicted PN40024 members from the respective subfamilies were included as representatives (Martin et al., 2010). The 533 proteins could be clustered into 111 representative sequences (Supplementary Data Sheet 4), indicted by the triangular nodes. Of the representative sequences, 24 *VviTPS-a*, 16 *VviTPS-b* and 7 *VviTPS-g* sequences were not connected to any other sequence indicating that they are unique.

The aforementioned results provides and overview of what is available in the respective networks, however, the data generated in this study is intended to be accessed and mined interactively. Networks can be accessed and downloaded through NDEx (Pratt et al., 2015): http://www.ndexbio.org/#/networkset/b90de24a-24fa-11ea-bb65-0ac135e8bacf?accesskey=c3cdbc1558016990ab78cab2e33cdc41b43c8333ea02799413ebb48f58abbe45. Supplementary Data Sheet 4 contains the representative VviTPS protein sequences, illustrated in Figure 9, and allow for the BLAST lookup for genes of interest using the “align two or more sequences” function of protein BLAST^3^. All nodes and edges in the respective networks are clickable and represent the entire collection of the data generated in this study. By interacting with the nodes and edges, a user can find the nearest functionally characterized protein, which includes metadata for NCBI accessions and nearest reference gene model, as predicted by Martin et al. (2010), as well as subfamily specific reaction mechanisms. It is therefore possible to query any new gene of interest against the current *VviTPS* gene family for the three diploid genomes and the PN40024 reference genome. We recommend viewing the networks on a local machine using Cytoscape (Shannon et al., 2003). A help document to guide users through this is made available with in Supplementary Data Sheet 4. The curated genomic, coding and protein sequences represented in the various networks are available as FASTA files in Supplementary Data Sheet 5.

## Discussion

The reference genome highlighted the extensive duplications and functional diversification of the *VviTPS* family (Martin et al., 2010). Although it was stated that paralogous genes are spread across the genome; identifying homologs were not possible due to the cultivar clone sequenced being near-homozygous (Jaillon et al., 2007). A typical approach to find paralogs entails a BLAST search to find a gene of interest, followed by locating it on the genome, usually through a genome browser. Although specialized gene families have been annotated for grapevine (Martin et al., 2010; Vannozzi et al., 2012), delayed incorporation of these annotations into the reference annotation (Canaguier et al., 2017) and limited visibility of these curations often result in an outdated annotation, most commonly 12x.v0, being used to interpret newly generated results (Grimplet and Cramer, 2019). For example, the web interface of Ensembl Plants (Howe et al., 2020) presents the most complete set of tools to analyze the grapevine genome, but still relies on the 12x.v0 assembly and annotation, limiting its use for the analysis of specialized gene families. Furthermore, the Nimblegen microarray platform utilized for numerous grapevine expression studies showed extensive probe ambiguities within the VviTPS family when using the 12x.v0 annotation, misrepresenting the expression patterns of *VviTPS* genes (Smit et al., 2019). The mapping of RNAseq reads to the aforementioned annotation presents a similar challenge, however, *de novo* assemblies of reads allow for more accurate profiling of *VviTPS* expression patterns (Da Silva et al., 2013; Venturini et al., 2013).

The link between *VviTPS* expression patterns and observed metabolites is therefore tenuous, requiring a critical re-evaluation. As we progress into a new generation of highly contiguous phased diploid genomes it is critical for expanded gene families involved in specialized metabolism to be accurately annotated. This is not only important from a wine aroma perspective but also from an ecophysiological perspective. Numerous terpenoids have been shown to provide important fitness advantages; this includes plant defense, abiotic and biotic stress and chemical signaling (reviewed in Pichersky and Raguso, 2018). The latter aspects will become increasingly important as we aim to breed hardier grapevines, with increased tolerance to climate fluctuations while maintaining sought after aromatic qualities.

The approach presented in this study was akin to that of a pangenome but utilizes a network for data visualization rather than a genome browser. Pangenomes typically focus on the differences and similarities between species, however, the genotypes presented here were expected to be highly similar due to it being closely related cultivars of the same species (Supplementary Figure 1). Although partial gene duplications were annotated (refer to the network illustrated by Figure 8), their evolutionary importance was not explored further. For the same reason the transposable elements proximal to *VviTPS* genes were excluded. Both of these aspects will become more relevant once the draft diploid genomes are assembled to chromosomes, allowing for in-depth analysis of collinearity and synteny. Nevertheless, the current unassembled genomes allow for a comparative analysis of the *VviTPS* family. The absolute position of *VviTPS* genes on the diploid genomes was therefore not a focus of this study, but rather how genes are related and how their putative function will impact the genetic potential of a genotype. This was possible due to the size of the highly contiguous diploid genome contigs having little to no overlap, in essence each representing a unique genomic region.

Function inference, is however, not purely based on sequence similarity due to the complexity of the carbocation cascades involved in enzyme catalytic mechanisms. The availability of various TPS crystal structures (Lesburg, 1997; Starks, 1997; Williams et al., 1998; Caruthers et al., 2000; Rynkiewicz et al., 2001; Shishova et al., 2007; Gennadios et al., 2009; Li et al., 2013) and functionally characterized enzymes, combined with quantum mechanical modeling (Bülow et al., 2000; Davis and Croteau, 2000; Gao et al., 2012; Miller and Allemann, 2012; Hong and Tantillo, 2014; Wedler et al., 2015; O’Brien et al., 2016; Durairaj et al., 2019), have contributed to elucidating how these cascades proceed in producing the thousands of naturally occurring terpene structures (Osbourn and Lanzotti, 2009; Buckingham et al., 2015).

Sequence identity and protein structure homology to experimentally characterized enzymes have been shown to be an effective approach to predict TPS reaction mechanisms (Degenhardt et al., 2009; Durairaj et al., 2019; Smit et al., 2019). However, this approach requires an extensive understanding of TPS reaction mechanisms, which is especially relevant when considering that the presence of a transcript does not necessarily correspond to a functional enzyme; and that enzyme mechanics can differ (within and between genotypes) due to mutations (Drew et al., 2015; Smit et al., 2019). The results generated in this study therefore provide a multi-genotype view of the *VviTPS* family, consisting of both gene annotation and functional predictions to disseminate and significantly expand on existing knowledge. The benefits of long-read sequencing, allowing for haplotype resolution, despite being unassembled, must be emphasized as it overcomes erroneous assembly of highly similar and duplicated gene regions that could not be resolved through short-read sequencing. The collection of interactive networks therefore provides a platform for studying this family in different grapevine genotypes and provides a novel approach for studying expanded gene families involved in specialized metabolism.

### The Grapevine *TPS-g* Family

Mapping of diploid contigs to the reference genome resulted in the identification of *VviTPS-g* members that localize to chr. 10. Two compounding factors made analyzing this family on the reference genome challenging: (1) chr. 10 is known to be highly discontiguous for the reference genome and (2); the PN40024 members of the *VviTPS-g* family are not placed on a chromosome, instead mapping to the chr. 00 pseudo-molecule (Martin et al., 2010; Canaguier et al., 2017). Analysis of the diploid genomes revealed that the contigs localizing to PN40024 chr. 10 had low RaGOO scores and high levels of heterozygosity (Supplementary Table 2 and Supplementary Data Sheet 3). This explains, to a large degree, the discontiguity of chr. 10 and the lack of VviTPS annotations. It may therefore be worthwhile (for the grapevine community) to consider remapping of the PN40024 short-reads to the phased diploid genomes in order to obtain a more contiguous chr. 10 for the reference genome. Nevertheless, the contiguity and size of the phased diploid contigs allowed us to overcome the aforementioned limitations, providing new insights into this important *VviTPS* subfamily (terpene alcohol biosynthesis) and its putative chromosome position.

The diploid genomes, as expected, show an increased number of putative *VviTPS-g* members (28–34 genes) with the function-specific clades being fairly conserved in gene number across the three genomes (Figure 7). The phylogenetic distribution within this subfamily, furthermore, highlights the limited number of functionally characterized enzymes that could be used to infer those that potentially contribute to the biosynthesis of terpene alcohols. Of the ten characterized *VviTPS-g* members, seven were characterized from Pinot Noir. Functional groupings in Figure 7 shows that dual substrate (GPP and FPP) enzymes capable of producing both linalool and nerolidol are overrepresented in all cultivars. Although a large clade of enzymes are predicted to use GGPP as well, resulting in (*E,E*)-geranyl linalool biosynthesis, the ability to use all three substrates *in planta* has not been reported. Subcellular compartmentalization of precursor pools (IPP and DMAPP) and regulation of prenyl substrate biosynthesis is tightly regulated, resulting in compartment-specific biosynthesis of terpenes (Wu et al., 2006; Heinig et al., 2013). Substrate specificity is thought to be affected by the active site, resulting in differential affinities to GPP, FPP and GGPP when enzymes are studied *in vitro* (Arimura et al., 2007; Pazouki et al., 2015). This was also shown for *PlTPS2* from *Phaseolus lunatus* (lima bean), however, *in planta* expression of this gene resulted in (*E,E*)-geranyl linalool and hemiterpene accumulation (Brillada et al., 2013). It is thus likely that the tri-substrate VviTPS-g clade is involved in (*E,E*)-geranyl linalool biosynthesis rather than (*3S*)-linalool and/or (*E*)-nerolidol biosynthesis.

The clade for geraniol biosynthesis had only two putatively functional proteins for CS, with CH and CR having 4 and 5, respectively (Figure 7). During winemaking, geraniol is readily metabolized by yeast during fermentation to form important wine odorants that, along with nerolidol and linalool derivatives, make up the core constituents of aromatic wines, often described as having a Muscat or “floral” aromas (King and Dickinson, 2000; Emanuelli et al., 2010). These transformations are facilitated by specific yeast genera that facilitate the reduction of the terpenoid or cleavage of glycosyl groups. The available substrate (cultivar-specific terpenoids) and vinification style will therefore directly influence the extent of floral aroma catalysis (Carrau et al., 2005; Cramer et al., 2014).

Furthermore, (*E*)-nerolidol and (*E,E*)-geranyl linalool are known precursors for the homoterpenes (E)-4,8-dimethyl-1,3,7-nonatriene (DMNT) and (E,E)- 4,8,12-trimethyltrideca-1,3,7,11-tetraene (TMTT), respectively. DMNT, is especially important from an ecological perspective due to it being emitted by various grapevine organs, with flower and leaf emissions linked to the attraction of the grapevine berry moth, *Lobesia botrana*, a major grapevine pest (Tasin et al., 2007). Recent efforts to alter the chemical emission profile of grapevine focused on overexpressing an (*E*)-beta-farnesene synthase, decreasing *L. botrana* attraction to grapevine (Salvagnin et al., 2018). The numerous *TPS-g* members annotated here therefore provide alternative targets to alter (*E,E*)-geranyl linalool, and by extension DMNT, biosynthesis.

### The *VviTPS-a* and *-b* Subfamilies: An Expanded Group With Specialized Reaction Mechanisms

The TPS-a and -b subfamilies are hypothesized to have evolved from diterpene synthases where the loss of the γ domain or transit peptide, coupled with changes in the active site, lead to neofunctionalization (Köksal et al., 2011a, b; Pazouki and Niinemets, 2016). This likely allowed for spatial-temporal regulation and specialization with vestigial functions explaining the ability to use multiple substrates *in vitro* (Pazouki and Niinemets, 2016).

Sesquiterpene synthases (VviTPS-a) represent the largest grapevine subfamily and are of special interest due to their ability to produce either a single terpene or a multitude of compounds. The diversity in sesquiterpenes is largely due to the extra double bond in FPP, compared to GPP, with subsequent isomerization to NPP resulting in further diversity. Currently accepted reaction mechanisms of plant sesquiterpene synthases (Durairaj et al., 2019) resulted in VviTPS-a members grouping according to which of these are used initial substrate (Figure 5). Premature quenching of the cyclization reaction, regardless of whether FPP or NPP is the initial substrate, results in the formation of acyclic sesquiterpenes, with two small but distinct clades (Figure 5) suggesting that there may be a distinction in substrate affinity. The isomerization step is rate-limiting (Cane et al., 1997; Miller and Allemann, 2012) which could explain why fewer enzymes are in the NPP clade, suggesting a possible specialized *in planta* function. It was previously shown that PN40024 had distinct clades for 1,10 and 1,11-cyclizations of FPP (Martin et al., 2010; Smit et al., 2019), however, the increased number of putative VviTPS-a proteins from the three diploid genomes added greater complexity to the conservation of enzyme mechanisms (Figures 5, 9). Three distinct clades were identified (Figure 5) with the functional enzymes of clades 2 and 3 sharing the same product profiles with a clear distinction between 1,10- and 1,11-cyclizations. The 1,10-cyclizations will proceed through the (*E,E*)-germacradienyl cation to either germacrene A or D as reactive intermediates. From the germacrene A intermediate, an alkyl migration of the eudesmyl cation will be necessary to explain the mechanism for enzymes in clade 2 of Figure 5A. A lack of such a migration and the presence of selinene-type synthases are congruent with the reaction mechanisms of enzymes in clade 1 of Figure 5A (Caruthers et al., 2000; Calvert et al., 2002; Christianson, 2017). Due to these subtle complexities in enzyme mechanics, VviTPS-a functional predictions is limited to initial substrate and first cyclization (Figure 5).

The PN40024 *VviTPS-b* subfamily consists of 45 loci, including pseudogenes, of which 19 were predicted to be functional. Seven of the nineteen have been functionally characterized, resulting in nine novel enzymes. The three phased diploid genomes contain between 37 and 65 *VviTPS-b* complete genes (fl- and d-ORF), excluding partial genes (Figure 6), providing an extended number of new *VviTPS-b* gene models. Although multiple reaction mechanism was identified for clades within the TPS-b subfamily, the overarching differences were between TPS-b Type I and II mechanisms. It was, however, noted that the single product Type II enzymes formed unique clades. This was also reported by Martin et al. (2010) where the two reaction types were bifurcated by sequences from other plants instead of a group of enzymes with no clear function, shown in Figure 6. The clades in Figure 6, indicate a conserved set of Type II enzymes that seemingly evolved to multi-product Type I enzymes. The clade of proteins associated with enzymes that accept FPP *in vitro* seems to be conserved, with its phylogenetic position supporting the specialization hypothesis (Pazouki and Niinemets, 2016).

## Conclusion

The availability of new genomic resources allowed for a comparative analysis of the *VviTPS* family, expanding on what the PN40024 genome offered. The resolution of haplotypes allowed for the identification of putative alleles with greater sequence contiguity, due to long-read sequencing, allowing for a comprehensive, and more complete annotation of this expanded gene family. Phylogenomic similarity and functional predictions greatly benefited from having expanded genotypic variation. This allowed for greater subfamily-specific functional predictions while addressing specific limitations on the reference genome, particularly the *VviTPS-g* subfamily. The data presented is not intended to be a static resource with the incorporation of inter-varietal and -species variations at genomic and single base-pair levels expected to improve the accuracy of functional predictions. The recent release of a phased *V. riparia* genome (Girollet et al., 2019) and nucleotide variation data from 472 *Vitis* species (Liang et al., 2019), specifically hold great promise for elucidating the impact that domestication and breeding had on *VviTPS* evolution, expansion and functionalization. Although the diploid genomes are currently available as draft assemblies, this limitation is expected to be addressed in the near future. Establishing congruency with the reference genome will most likely require a critical re-evaluation of the PN40024 genome assembly to address the numerous limitations regarding its completeness and contiguity. The utilization of networks to show relatedness of *VviTPS* genes at the genomic, coding and protein sequence levels within and between cultivars provides a novel, valuable and interactive resource. This resource is intended to provide a starting platform from which genotypic variation can be explored and expanded on to characterize the *VviTPS* family further, while providing a blueprint for future comparative analyses of specialized gene families.

## Data Availability Statement

The datasets generated for this study can be found in the NDEx repository http://www.ndexbio.org/#/networkset/b90de24a-24fa -11ea-bb65-0ac135e8bacf?accesskey=c3cdbc1558016990ab78cab 2e33cdc41b43c8333ea02799413ebb48f58abbe45.

## Author Contributions

SS, MV, and PY conceptualized the study. SS performed all computational analyses, gene annotations and drafted the initial manuscript. All authors contributed to the final manuscript.

## Conflict of Interest

The authors declare that the research was conducted in the absence of any commercial or financial relationships that could be construed as a potential conflict of interest.

## Footnotes

1 https://urgi.versailles.inra.fr/Species/Vitis/Annotations

2 www.vivc.de (accessed November 18, 2019)

3 https://blast.ncbi.nlm.nih.gov/Blast.cgi
