# Supplementary figures and images for: Comparative (Within Species) Genomics of the Vitis vinifera L. Terpene Synthase Family to Explore the Impact of Genotypic Variation Using Phased Diploid Genomes
Source: Front Genet. 2020 May 5;11:421. doi: 10.3389/fgene.2020.00421 (PMC7216305; doi:10.3389/fgene.2020.00421)

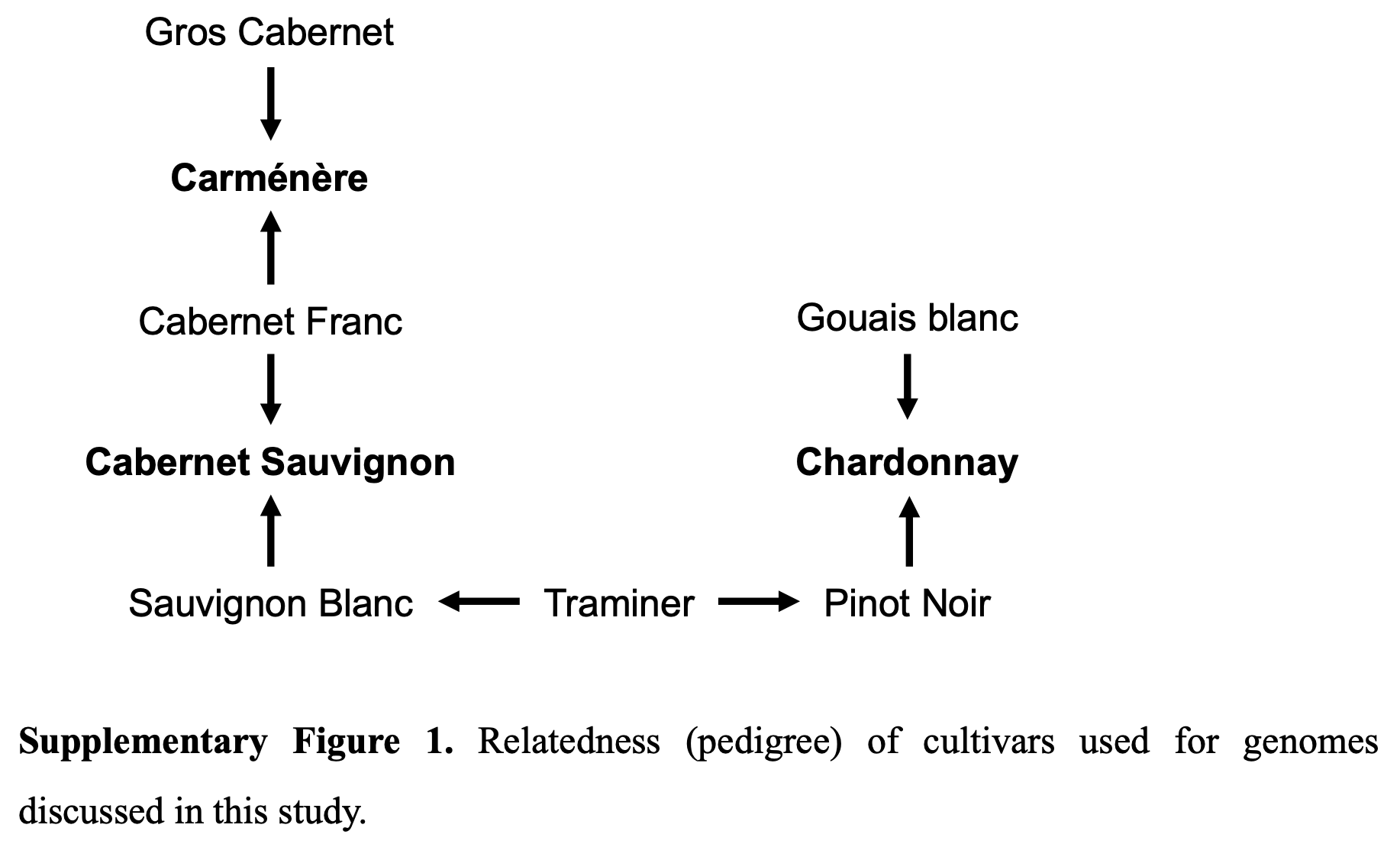

Supplement: Supplementary file 1 [file Image_1.TIFF]

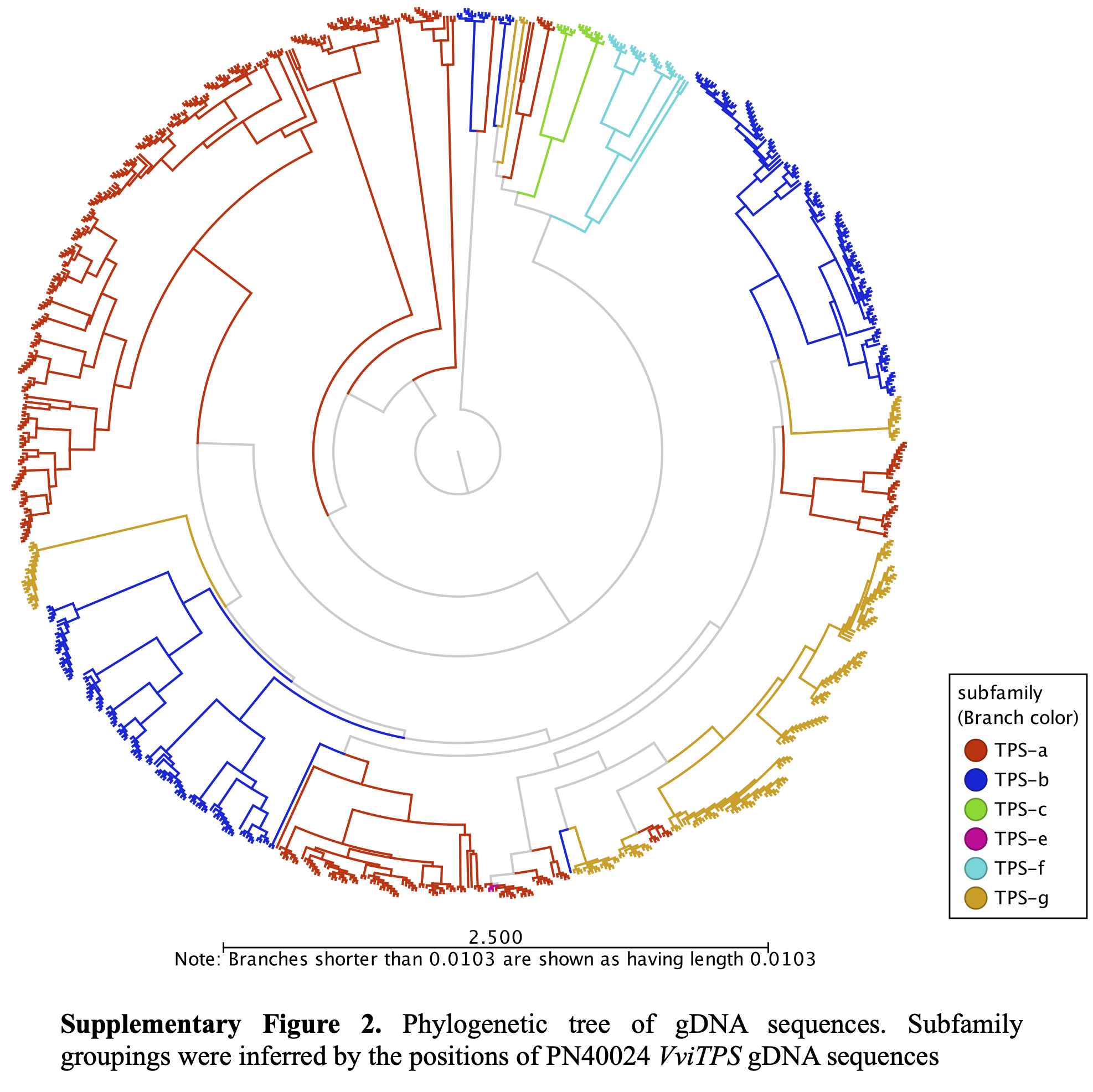

Supplement: Supplementary file 2 [file Image_2.TIFF]

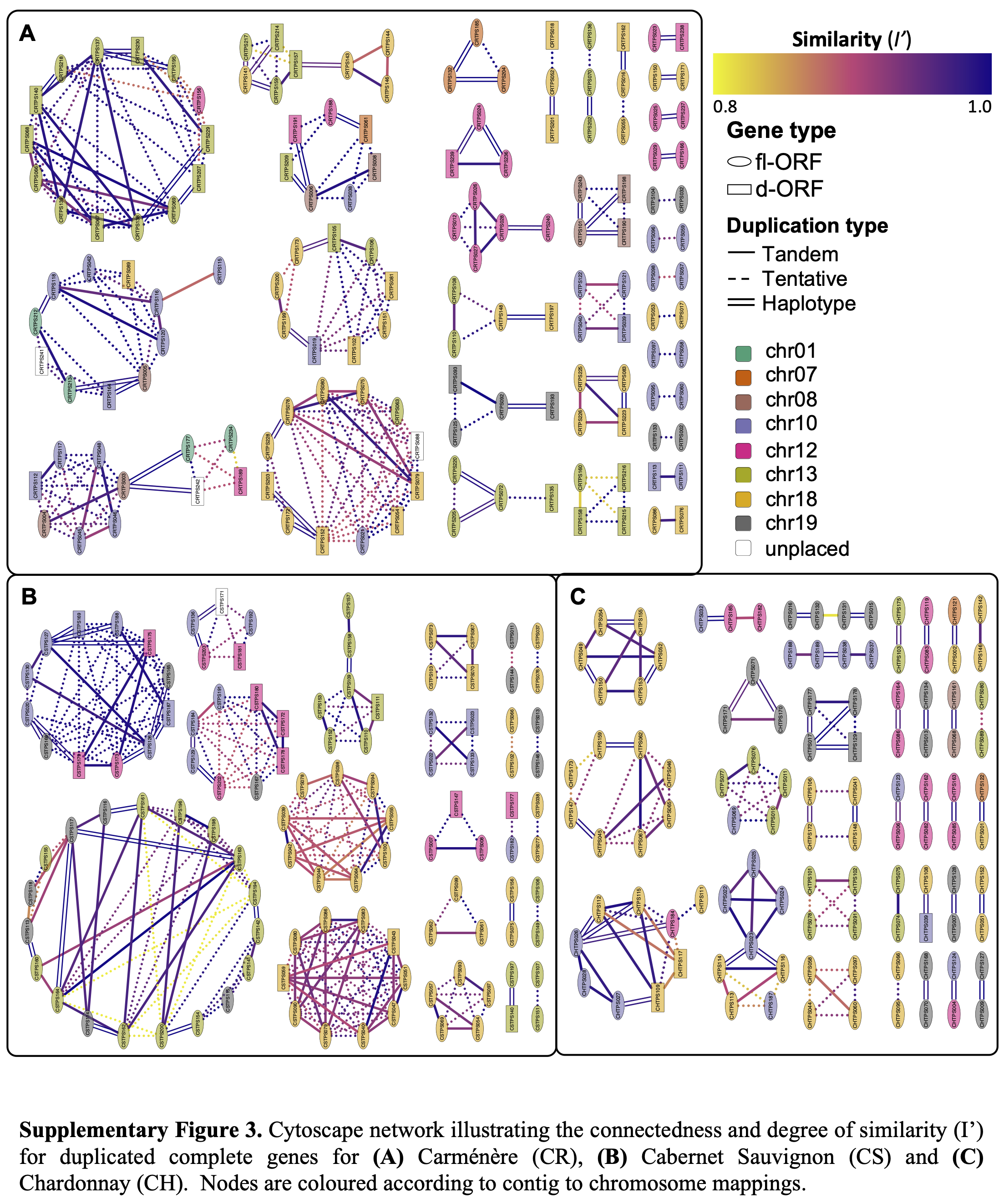

Supplement: Supplementary file 3 [file Image_3.TIFF]

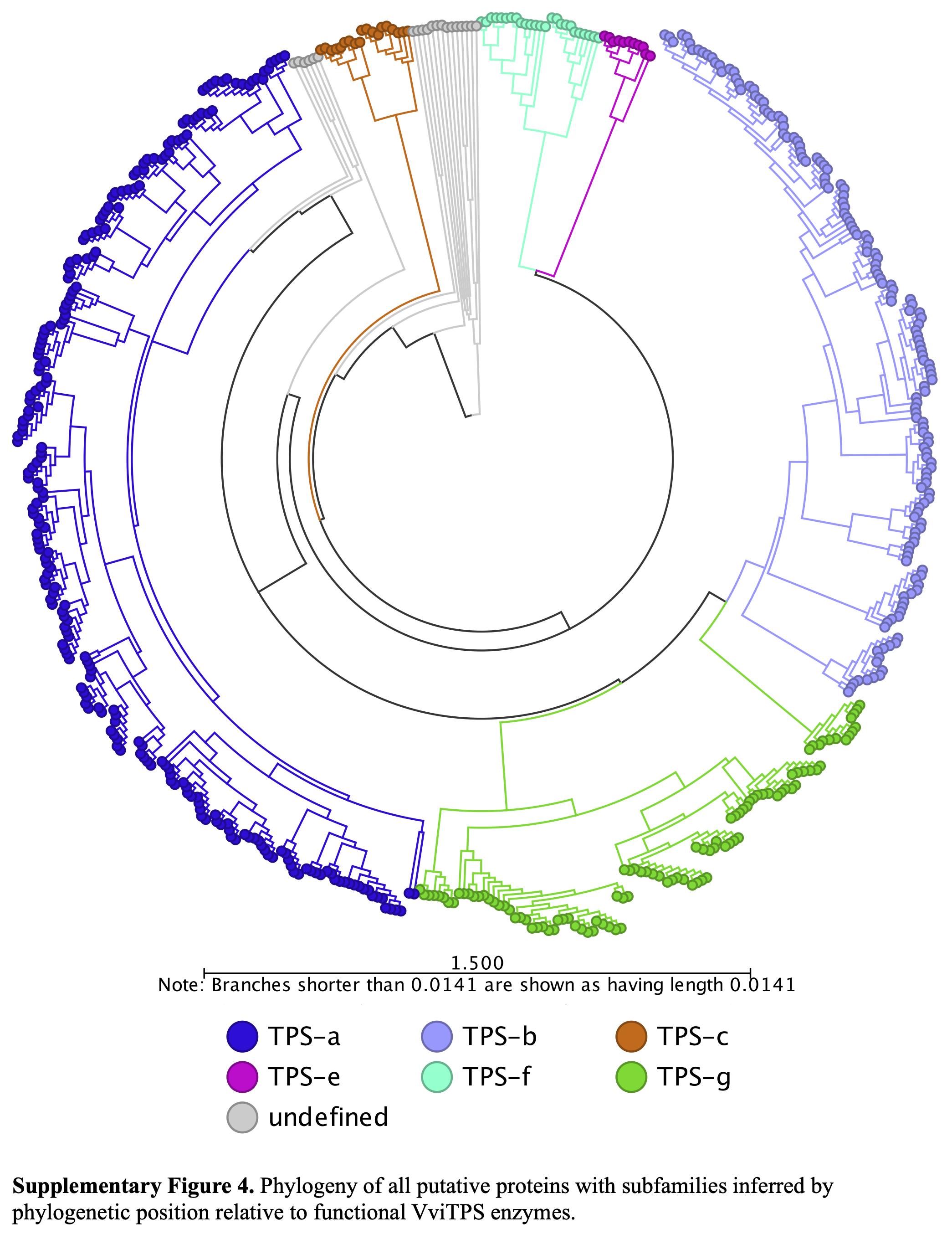

Supplement: Supplementary file 4 [file Image_4.TIFF]
