# Supplementary material for: Comparative (Within Species) Genomics of the Vitis vinifera L. Terpene Synthase Family to Explore the Impact of Genotypic Variation Using Phased Diploid Genomes
Source: Front Genet. 2020 May 5;11:421. doi: 10.3389/fgene.2020.00421 (PMC7216305; doi:10.3389/fgene.2020.00421)
Supplement: Supplementary file 5 [file Data_Sheet_1.PDF]

Supplementary Data Sheet 1

```
# The shell script below is an example shows how Exonorate est2genome
mapping was performed
# The script was adapted from the wiki posted by alvaralmstedt on github at
https://github.com/alvaralmstedt/Tutorials/wiki/From-exonerate-to-igv:-A-
story-about-GFF

############################################
# Step 1: Perform the exonerate analyses
############################################

# to execute the shell script on a Mac, open your terminal and run:
# sh /path_to/example_script.sh

# you need to specify paths for the following
# --query
# --target
# output

echo "Starting exonerate e2g at:"
date
wait

# chunk 1
exonerate -m est2genome --percent 90 --maxintron 3000 --showtargetgff yes -
-showalignment no --ryo ">%qi length=%ql alnlen=%qal\n>%ti length=%tl
alnlen=%tal\n" --targetchunkid 1 --targetchunktotal 8 --query
path_to/query.fasta --target path_to/target.fasta >
path_to/output_e2g_chunk1.output &

# chunk n
# repeat this for the number of chunks (8 in the example above) you
specified and change the output chunk number to match the targetchunkid
number

wait
echo "All done with e2g at:"
date

############################################
# Step 2: Concatenate output files
############################################

cat * > output_e2g.gff
# This will concatenate all output chunks in the directory to one GFF file.
This file can be imported as is into CLC Main Workbench 7
```
