# Supplementary material for: Comparative (Within Species) Genomics of the Vitis vinifera L. Terpene Synthase Family to Explore the Impact of Genotypic Variation Using Phased Diploid Genomes
Source: Front Genet. 2020 May 5;11:421. doi: 10.3389/fgene.2020.00421 (PMC7216305; doi:10.3389/fgene.2020.00421)
Supplement: Supplementary file 6 [file Data_Sheet_2.PDF]

Supplementary Data Sheet 2

```
# STEP 1: create your databases

mmseqs createdb <path/to/file> <DB name>
# specify the path to the fasta file you want to make a database of
# replace queryDB with a name for the database
# note that the DB will be created in the current working directory

# STEP 2: perform the clustering

# mmseqs cluster <DB> <DB_clu> tmp
# DB = database file
# DB_clu =  the output file
# tmp is the the tmp file where all the behind the scene stuff is dumped
# the following parameters were used in this study

mmseqs cluster <DB> <DB_clu> tmp --cov-mode 0 -c 0.85 -e 0.00001 --min-seq-
id 0.75 --cluster-mode 2

# To extract the clustering output as a text file
mmseqs createtsv <i:queryDB> [<i:targetDB>] <i:resultDB> <o:tsvFile>
[options]

# To extract the representative sequence for each cluster
mmseqs result2repseq <i:sequenceDB> <i:resultDB> <o:sequenceDb> [options]
mmseqs result2flat <i:queryDB> <i:targetDB> <i:resultDB> <o:fastaDB>
[options]
```
