# Supplementary material for: Comparative (Within Species) Genomics of the Vitis vinifera L. Terpene Synthase Family to Explore the Impact of Genotypic Variation Using Phased Diploid Genomes
Source: Front Genet. 2020 May 5;11:421. doi: 10.3389/fgene.2020.00421 (PMC7216305; doi:10.3389/fgene.2020.00421)
Supplement: Supplementary file 7 [file Data_Sheet_3.PDF]

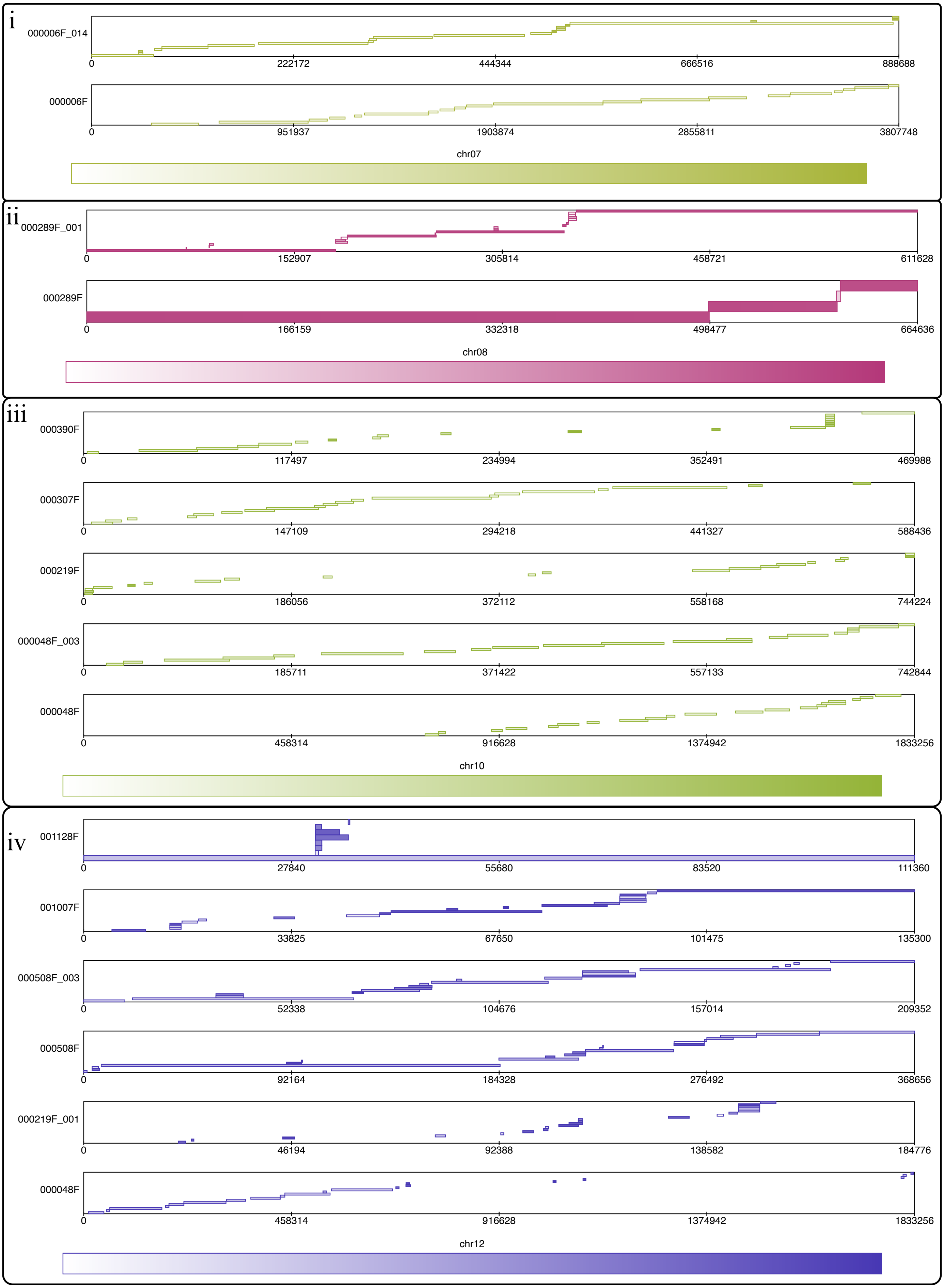

Supplementary Data Sheet 1A. Alvis contig alignments for Chardonnay (CH) to PN40024 chr. 07 (i), chr. 08 (ii), chr. 10 (iii) and chr12 (iv)

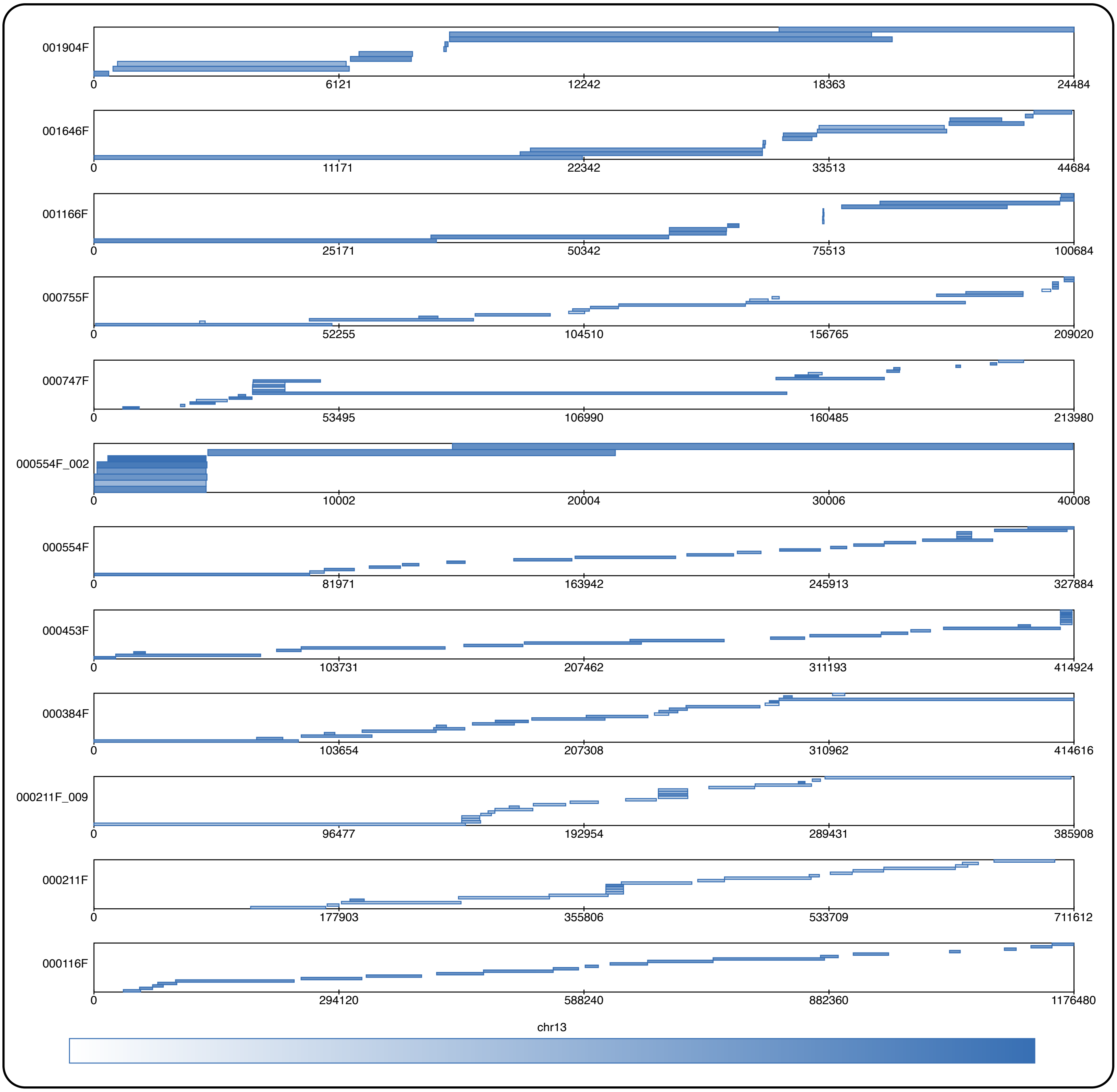

Supplementary Data Sheet 1B. Alvis contig alignments for Chardonnay (CH) to PN40024 chr. 13

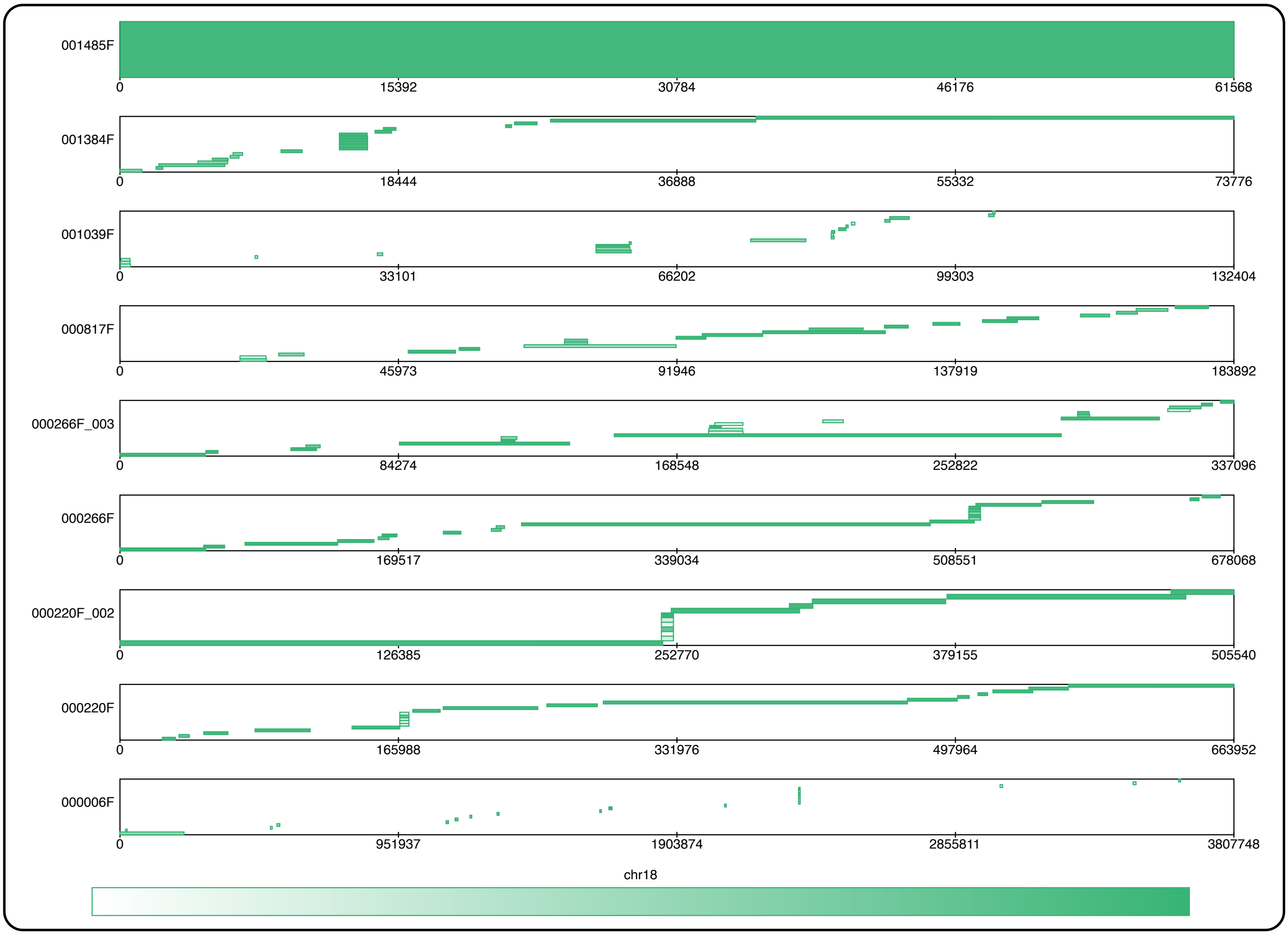

Supplementary Data Sheet 1C. Alvis contig alignments for Chardonnay (CH) to PN40024 chr. 18

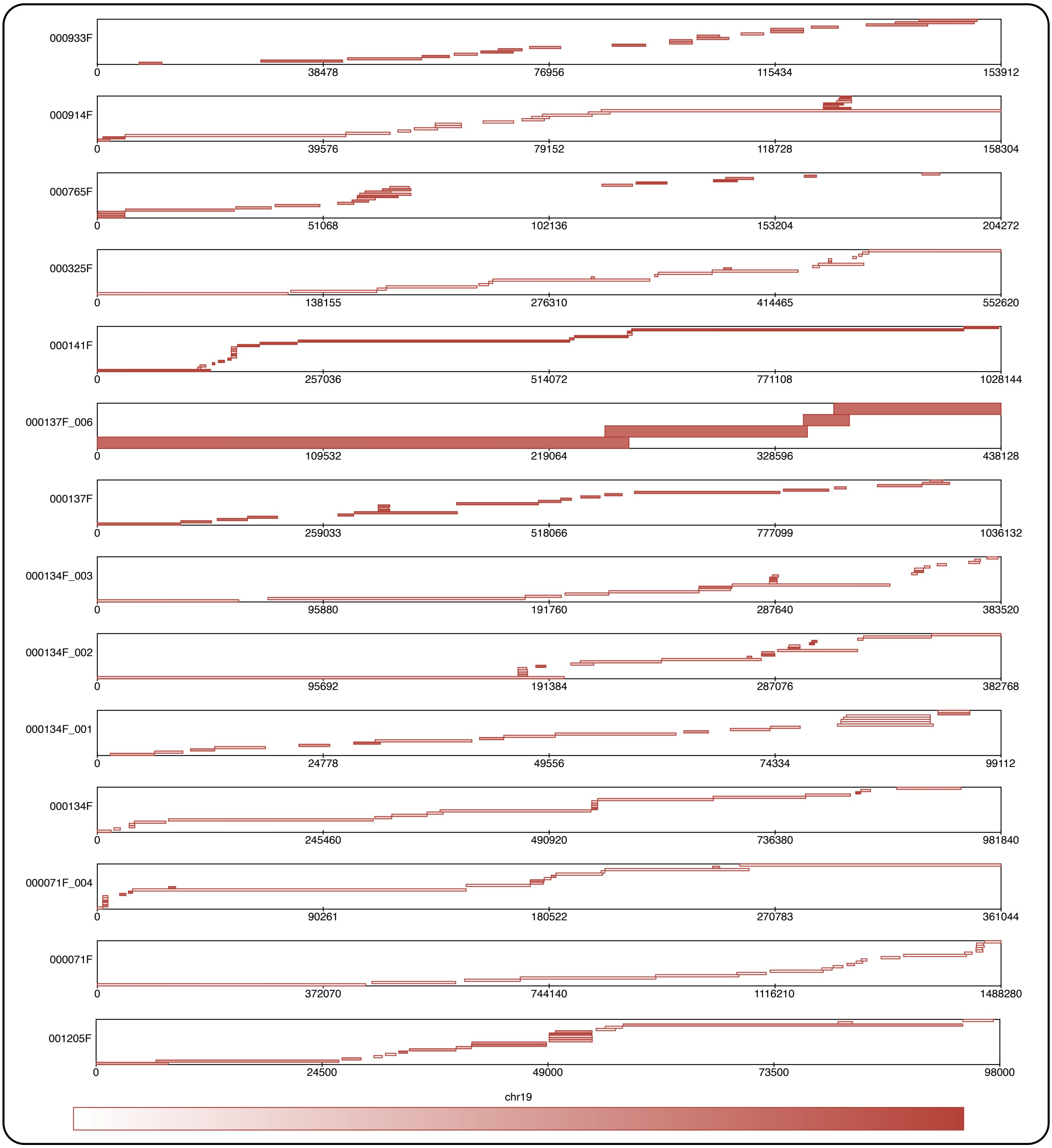

Supplementary Data Sheet 1D. Alvis contig alignments for Chardonnay (CH) to PN40024 chr. 19

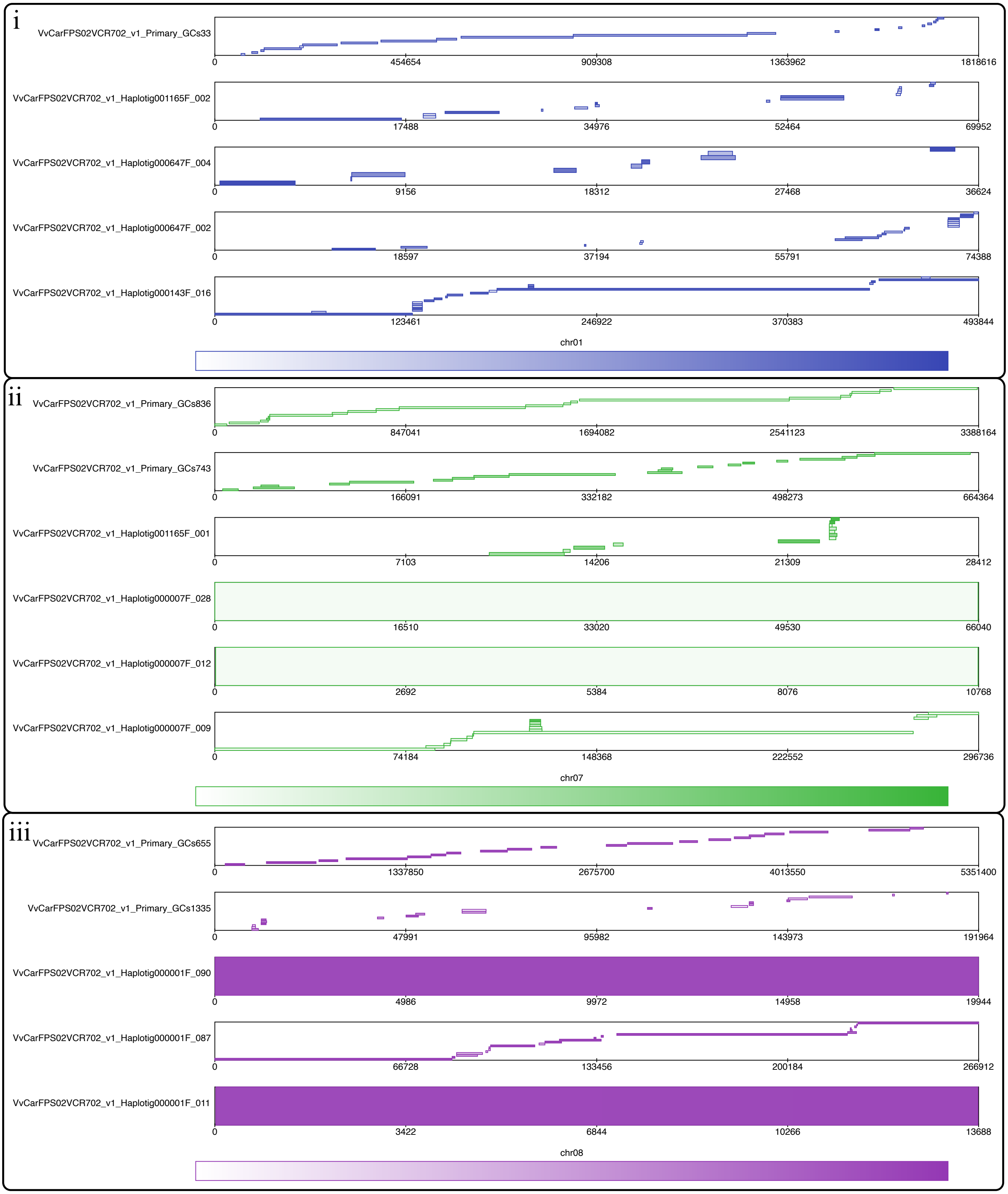

Supplementary Data Sheet 1E. Alvis contig alignments for Carménère (CR) to PN40024 chr. 01 (i), chr. 07 (ii) and chr. 08 (iii)

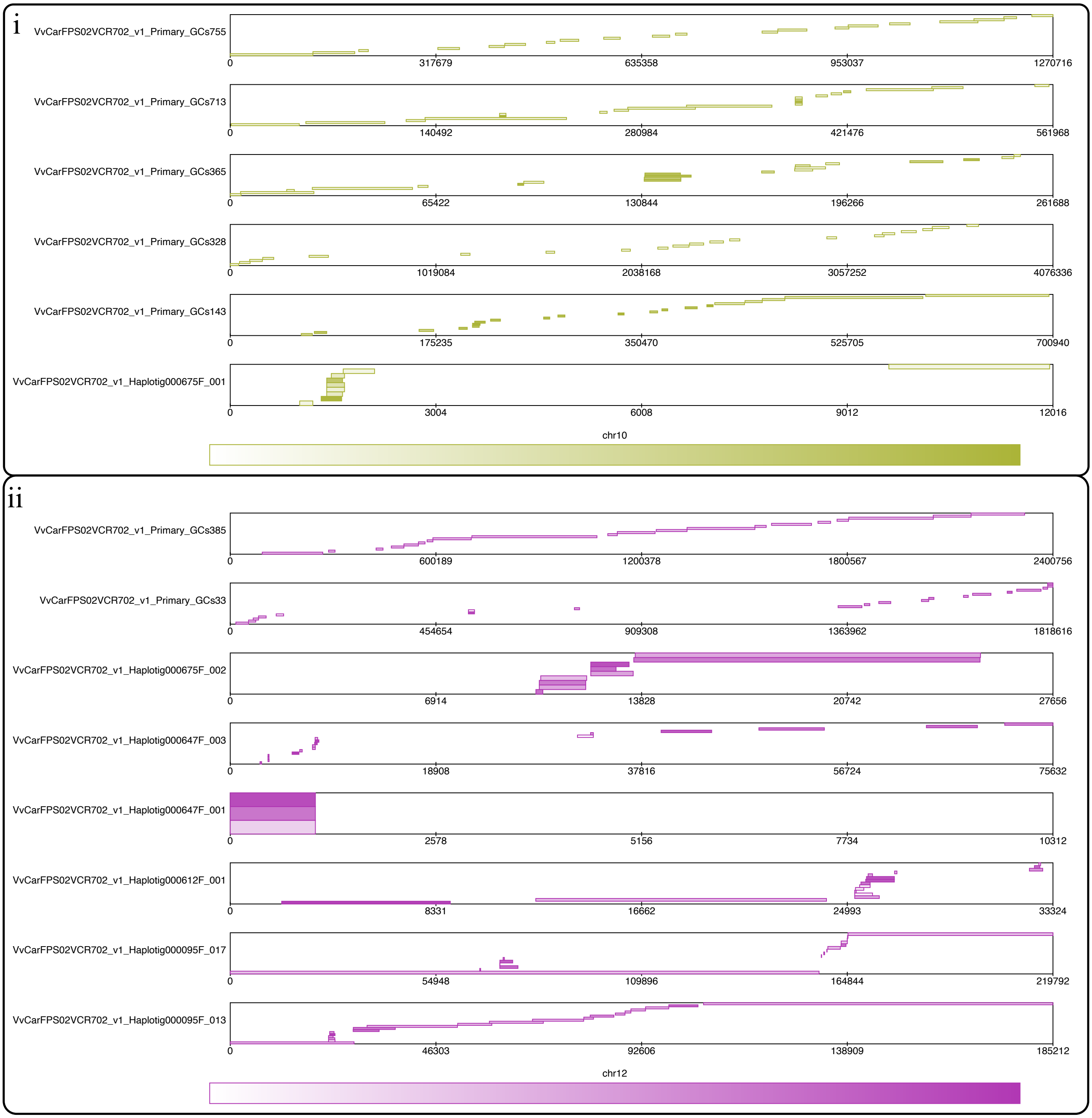

Supplementary Data Sheet 1F. Alvis contig alignments for Carménère (CR) to PN40024 chr. 10 (i) and chr. 12 (ii)

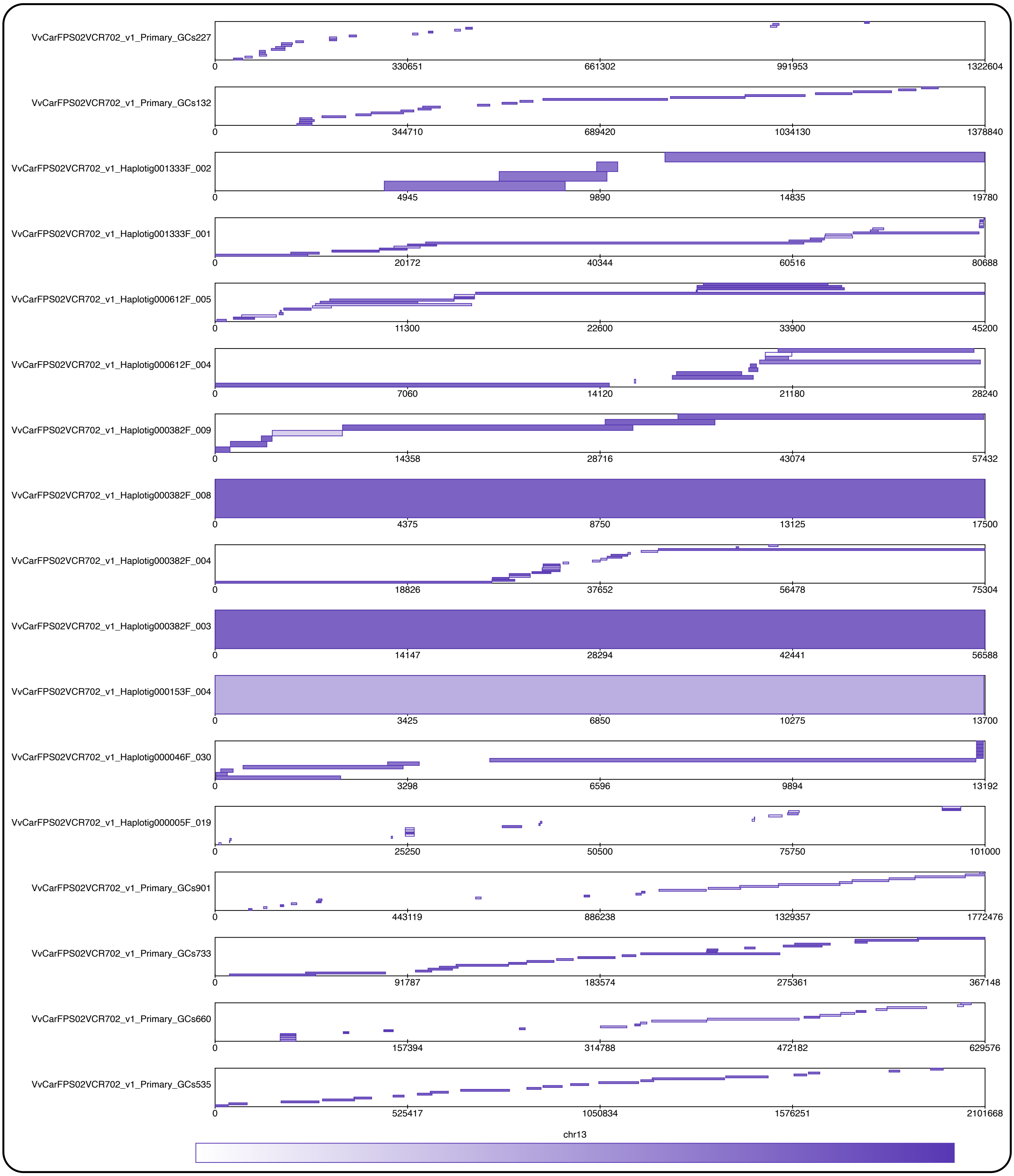

Supplementary Data Sheet 1G. Alvis contig alignments for Carménère (CR) to PN40024 chr. 13

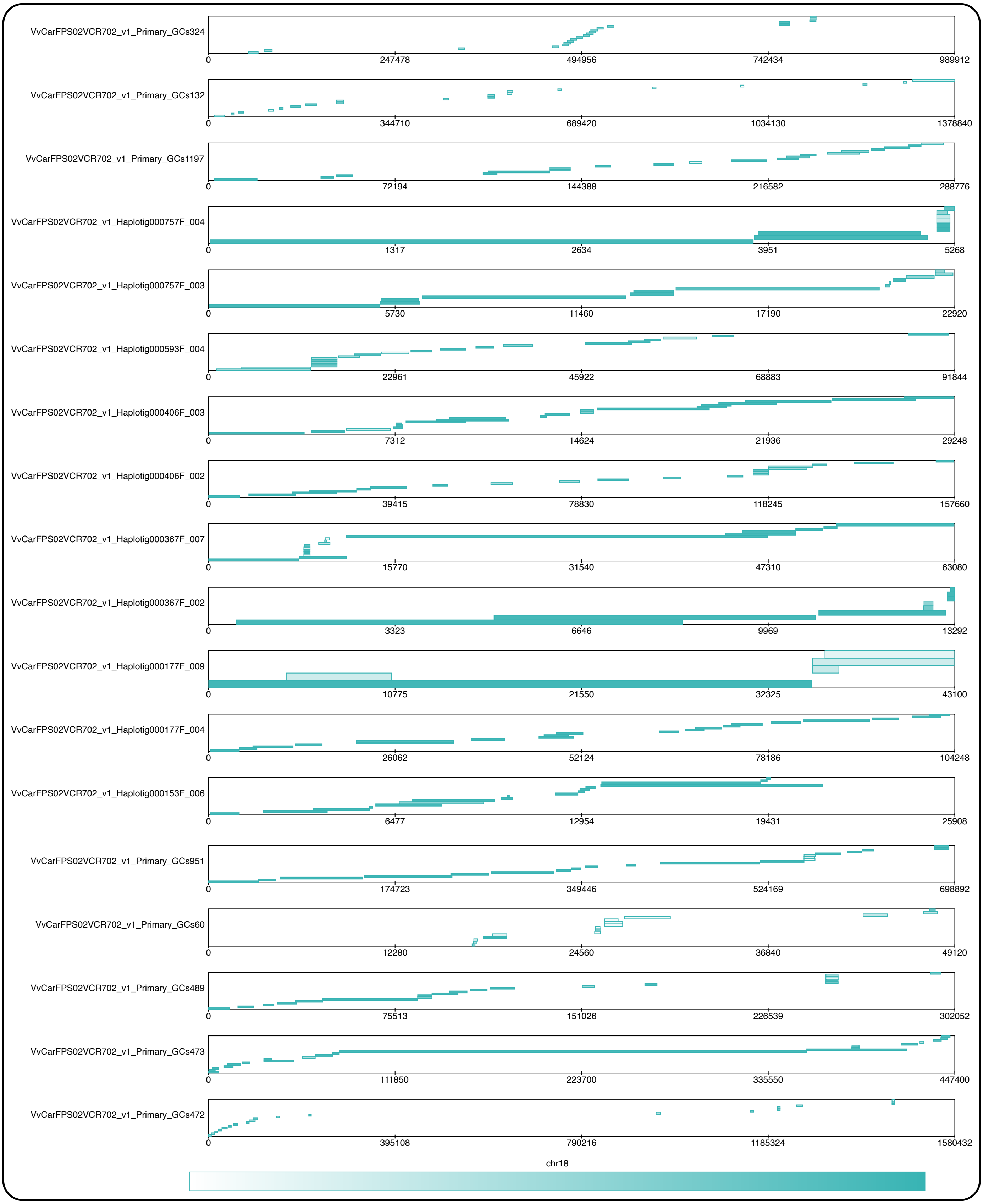

Supplementary Data Sheet 1H. Alvis contig alignments for Carménère (CR) to PN40024 chr. 18.

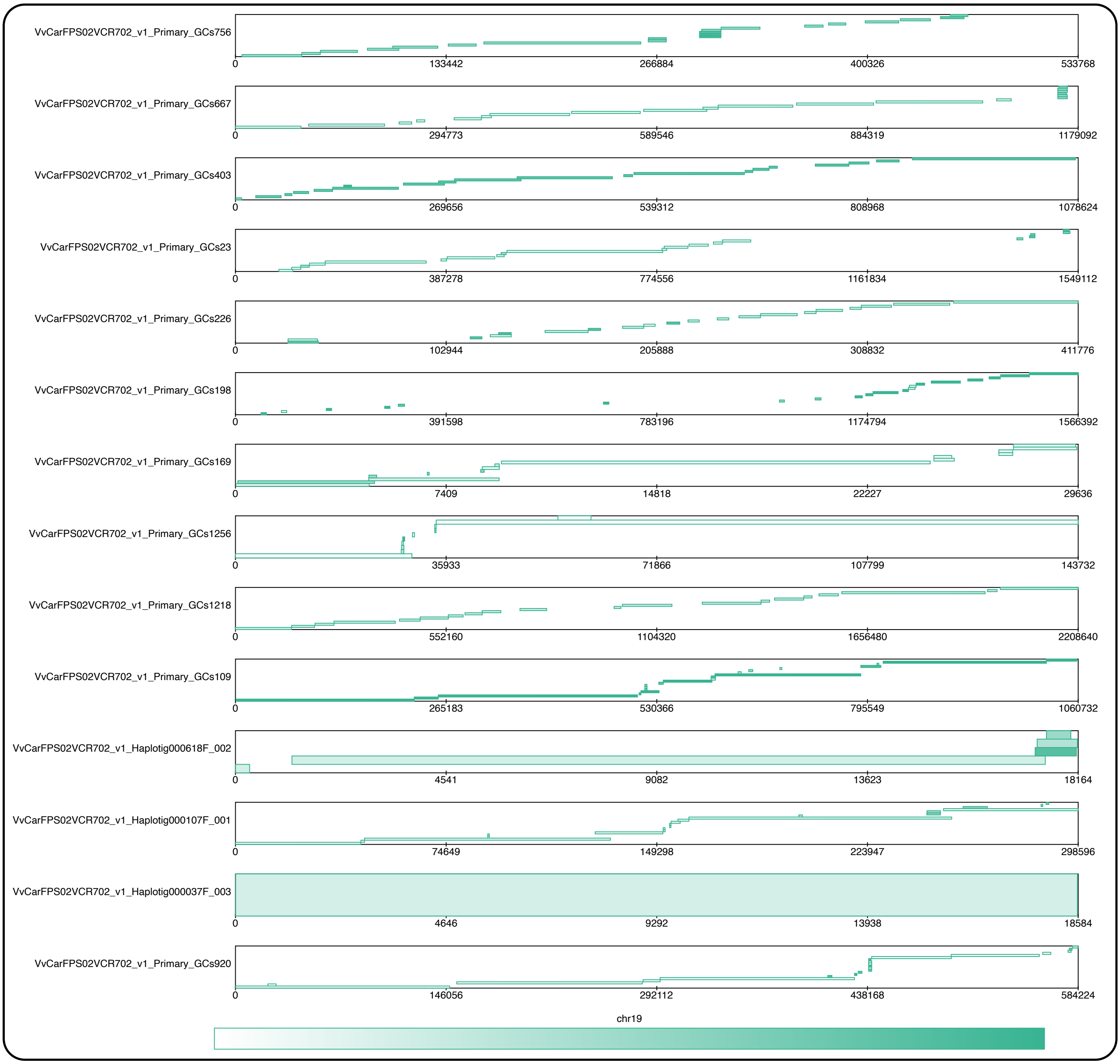

Supplementary Data Sheet 1I. Alvis contig alignments for Carménère (CR) to PN40024 chr. 19

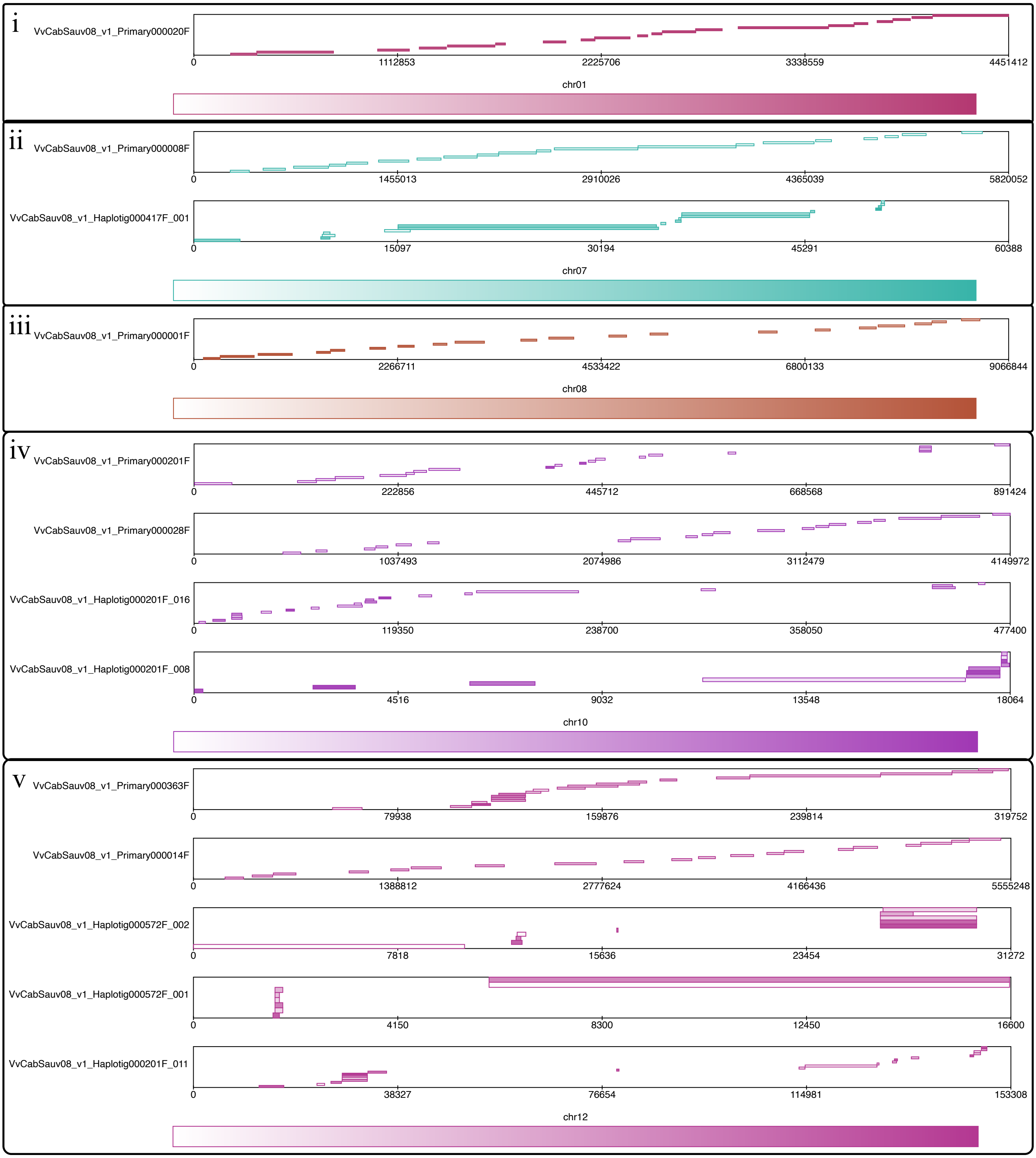

Supplementary Data Sheet 1J. Alvis contig alignments for Cabernet Sauvignon (CS) to PN40024 chr. 01, (i), chr. 07 (ii), chr. 08 (iii), chr. 10 (iv), chr. 12 (v)

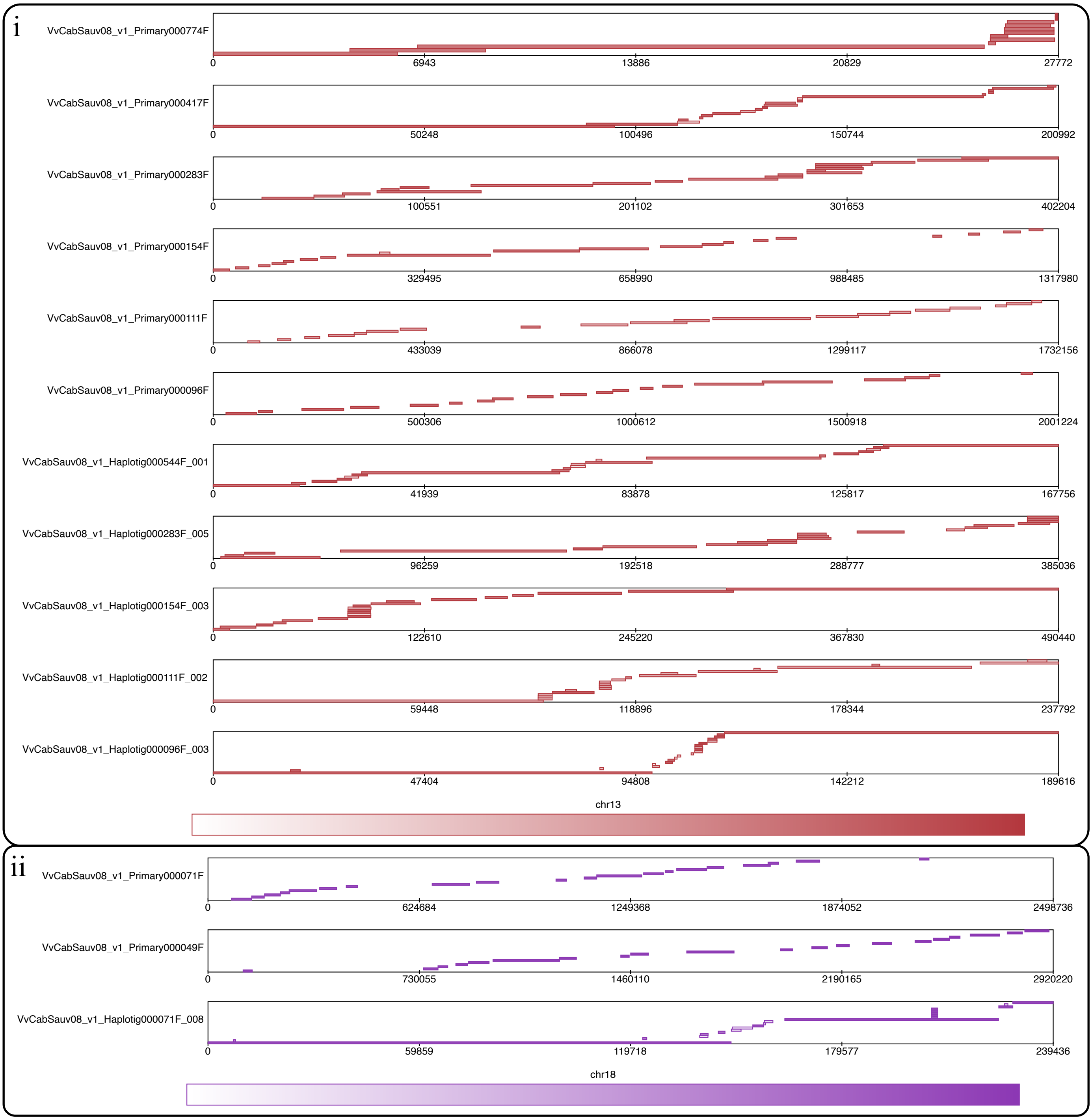

Supplementary Data Sheet 1K. Alvis contig alignments for Cabernet Sauvignon (CS) to PN40024 chr. 13, (i) and chr. 18 (ii).

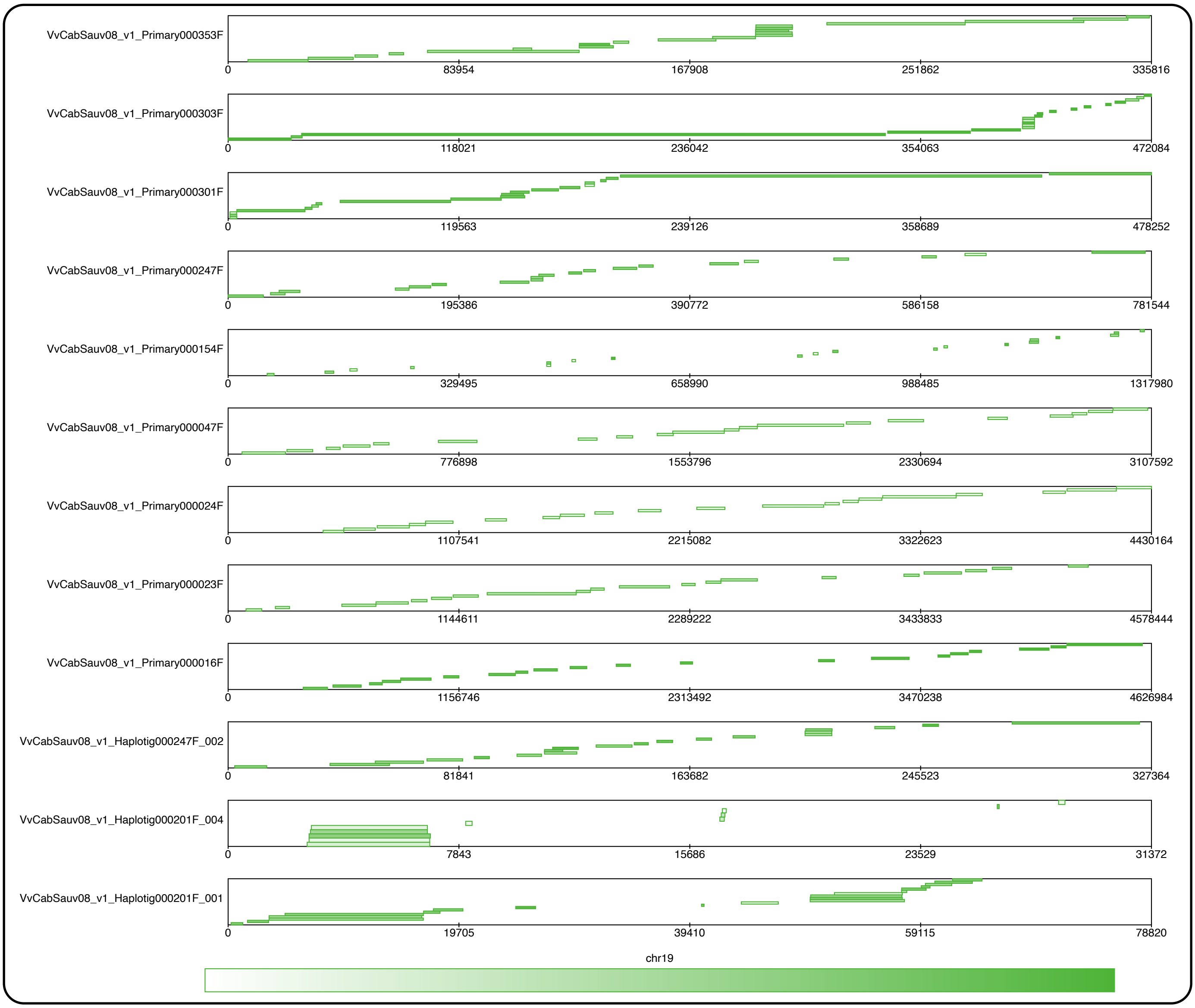

Supplementary Data Sheet 1L. Alvis contig alignments for Cabernet Sauvignon (CS) to PN40024 chr. 19
