# Supplementary material for: Comparative (Within Species) Genomics of the Vitis vinifera L. Terpene Synthase Family to Explore the Impact of Genotypic Variation Using Phased Diploid Genomes
Source: Front Genet. 2020 May 5;11:421. doi: 10.3389/fgene.2020.00421 (PMC7216305; doi:10.3389/fgene.2020.00421)
Supplement: Supplementary file 8 [file Data_Sheet_4.ZIP › Supplementary Data Sheet 4/Instructions.pdf]

Instructions to query genes of interest against the network

How to query the representative proteins using BLAST

1. Open blastp: https://blast.ncbi.nlm.nih.gov/Blast.cgi
2. Select "Align two or more sequences"
3. Upload file "VviTPS_subfam_prot_rep.fa" under the subject sequence section
4. Blast your protein sequence(s) of interest.

Using the VviTPS compendium

In order to fully engage with the networks we recommend that they be downloaded from NDeX using the link below and viewed in Cytoscape

Accessed the networks using the link below:

http://www.ndexbio.org/#/networkset/55b44ed9-0a9b-11ea-bb65-0ac135e8bacf?accesskey=b7ecfff59fb12ddc1de771abcabf3b84b08a62ef0dba8d65a4488f79b509a4f5

For online exploration of the network:
Go to the search bar under the network and paste your query ID from the blast output followed by a *

example: CHTPS001*

Under type select 2-step neighbourhood

Run Query

To access all the metadata in tabular form, please click on the “Table” button at the bottom right of the NDEx page.
