# Supplementary material for: Comparative (Within Species) Genomics of the Vitis vinifera L. Terpene Synthase Family to Explore the Impact of Genotypic Variation Using Phased Diploid Genomes
Source: Front Genet. 2020 May 5;11:421. doi: 10.3389/fgene.2020.00421 (PMC7216305; doi:10.3389/fgene.2020.00421)
Supplement: Supplementary file 10 [file Table_1.DOCX]

**Supplementary Table 1.** Protein sequences used to infer enzyme mechanisms

| Gene ID | Accession | Martin Gene Model | Reference |
| --- | --- | --- | --- |
| VvGwECar1 | HM807373 | VviTPS01 | (Martin et al., 2010) |
| VvGwECar2 | HM807374 | VviTPS27 | (Martin et al., 2010) |
| VvGwECar3 | HM807375 | VviTPS02 | (Martin et al., 2010) |
| VvPNECar1 | HM807402 | VviTPS02 | (Martin et al., 2010) |
| VvPNECar2 | HM807403 | VviTPS13 | (Martin et al., 2010) |
| VvGwGerA | HQ326230 | VviTPS01 | (Martin et al., 2010) |
| VvGwaBer | HM807376 | VviTPS10 | (Martin et al., 2010) |
| VvGwGerD | HM807377 | VviTPS07 | (Martin et al., 2010) |
| VvPNGerD | HM807378 | VviTPS15 | (Martin et al., 2010) |
| VvCSaFar | HM807379 | VviTPS20 | (Martin et al., 2010) |
| VvGwgCad | HM807380 | VviTPS08 | (Martin et al., 2010) |
| VvPNbCur | HM807381 | VviTPS30 | (Martin et al., 2010) |
| VvPNSesq | HM807404 | VviTPS12 | (Martin et al., 2010) |
| VvPNaZin | HM807405 | VviTPS14 | (Martin et al., 2010) |
| VvPNSeInt | HM807406 | VviTPS24 | (Martin et al., 2010) |
| VvPNCuCad | HM807407 | VviTPS26 | (Martin et al., 2010) |
| VvPNaHum | HM807408 | VviTPS11 | (Martin et al., 2010) |
| VvPNEb2epi Car | HM807409 | VviTPS21 | (Martin et al., 2010) |
| VvGuaS | HM807406 | N/A | (Drew et al., 2015) |
| VvVal | AY561843 | N/A | (Lücker et al., 2004) |
| VvGerD | AY561842 | N/A | (Lücker et al., 2004) |
| VvValCS | FJ696653 | N/A | (Martin et al., 2009) |
| VvGwaPhe | HM807382 | VviTPS45 | (Martin et al., 2010) |
| VvPNaPin1 | HM807383 | VviTPS44 | (Martin et al., 2010) |
| VvPNaPin2 | HM807384 | VviTPS44 | (Martin et al., 2010) |
| VvGwbOci | HM807385 | VviTPS34 | (Martin et al., 2010) |
| VvCSbOci | HM807386 | VviTPS35 | (Martin et al., 2010) |
| VvCSbOciM | HM807387 | VviTPS38 | (Martin et al., 2010) |
| VvGwbOciF | HM807388 | VviTPS47 | (Martin et al., 2010) |
| VvCSbOciF | HM807389 | VviTPS47 | (Martin et al., 2010) |
| VvPNRLin | HM807390 | VviTPS31 | (Martin et al., 2010) |
| VvPNLinNer1 | HM807391 | VviTPS54 | (Martin et al., 2010) |
| VvPNLinNer2 | HM807392 | VviTPS56 | (Martin et al., 2010) |
| VvCSLinNer | HM807393 | VviTPS56 | (Martin et al., 2010) |
| VvPNLNGl1 | HM807394 | VviTPS57 | (Martin et al., 2010) |
| VvPNLNGl2 | HM807395 | VviTPS63 | (Martin et al., 2010) |
| VvPNLNGl3 | HM807396 | VviTPS58 | (Martin et al., 2010) |
| VvPNLNGl4 | HM807397 | VviTPS61 | (Martin et al., 2010) |
| VvGwGer | HM807398 | VviTPS52 | (Martin et al., 2010) |
| VvCSGer | HQ326231 | VviTPS52 | (Martin et al., 2010) |
| VvPNGer | HM807399 | VviTPS52 | (Martin et al., 2010) |
| VvCSENerGl | HM807400 | N/A | (Martin et al., 2010) |
| VvPNENerGl | HM807401 | N/A | (Martin et al., 2010) |
| VviMATPS10 | QBL52481.1 |  | (Smit et al., 2019) |
