# Supplementary material for: Comparative (Within Species) Genomics of the Vitis vinifera L. Terpene Synthase Family to Explore the Impact of Genotypic Variation Using Phased Diploid Genomes
Source: Front Genet. 2020 May 5;11:421. doi: 10.3389/fgene.2020.00421 (PMC7216305; doi:10.3389/fgene.2020.00421)
Supplement: Supplementary file 11 [file Table_2.DOCX]

**Supplementary Table 2.** Average RaGOO scores per chromosome for the three cultivars assembled to PN40024 12x.v2

| **Chr.** | **Cv.** | **Location Score** | **Orientation Score** | **Grouping Score** |
| --- | --- | --- | --- | --- |
| chr01 | CS | 9% | 98% | 87% |
|  | CR | 15% | 97% | 53% |
| chr07 | CS | 9% | 94% | 74% |
|  | CR | 47% | 95% | 82% |
|  | CH | 9% | 99% | 93% |
| chr08 | CS | 22% | 99% | 93% |
|  | CR | 45% | 92% | 82% |
|  | CH | 4% | 99% | 97% |
| chr10 | CS | 5% | 70% | 65% |
|  | CR | 21% | 66% | 76% |
|  | CH | 3% | 59% | 74% |
| chr12 | CS | 25% | 89% | 57% |
|  | CR | 30% | 92% | 67% |
|  | CH | 4% | 88% | 68% |
| chr13 | CS | 19% | 87% | 83% |
|  | CR | 36% | 91% | 70% |
|  | CH | 16% | 86% | 74% |
| chr18 | CS | 5% | 97% | 79% |
|  | CR | 12% | 86% | 64% |
|  | CH | 14% | 91% | 76% |
| chr19 | CS | 12% | 85% | 64% |
|  | CR | 21% | 97% | 75% |
|  | CH | 11% | 91% | 77% |
| **Average** |  | **17%** | **89%** | **75%** |
